# A Probabilistic Model of the Germinal Center Reaction

**DOI:** 10.3389/fimmu.2019.00689

**Published:** 2019-04-03

**Authors:** Marcel Jan Thomas, Ulf Klein, John Lygeros, María Rodríguez Martínez

**Affiliations:** ^1^IBM Research Zürich, Rüschlikon, Switzerland; ^2^ETH Zürich, Automatic Control Laboratory, Zurich, Switzerland; ^3^Experimental Haematology, Leeds Institute of Medical Research at St. James's, University of Leeds, Leeds, United Kingdom

**Keywords:** germinal center, B cell, memory B cell differentiation, plasma cell differentiation, asymmetric centroblast division

## Abstract

Germinal centers (GCs) are specialized compartments within the secondary lymphoid organs, where B cells proliferate, differentiate, and mutate their antibody genes. Upon exit from the GC, B cells terminally differentiate into plasma cells or memory B cells. While we have a good comprehension of plasma cell differentiation, memory B cell differentiation is still incompletely understood. In this paper, we extend previous models of the molecular events underlying B cell differentiation with new findings regarding memory B cell formation, and present a quantitative stochastic model of the intracellular and extracellular dynamics governing B cell maturation and exit from the GC. To simulate this model, we develop a novel extension to the Gillespie algorithm that enables the efficient stochastic simulation of the system, while keeping track of individual cell properties. Our model is able to explain the dynamical shift from memory B cell to plasma cell production over the lifetime of a GC. Moreover, our results suggest that B cell fate selection can be explained as a process that depends fundamentally on antigen affinity.

## 1. Introduction

Germinal centers (GCs) are temporary but distinct structures in the lymph nodes or the spleen, where B cell affinity maturation and differentiation into plasma cells (PCs) and memory B cells (MBCs) takes place (1–4). GC dynamics mimic evolution. First, GC B cells rapidly divide and modify their immunoglobulin variable region (IgV) genes of the B cell receptor (BCR) through somatic hypermuation (SHM) and class switch recombination (CSR), which binds foreign antigen. GC B cells are next selected according to the affinity of their BCRs to antigen. B cells displaying low affinity to antigen, for instance those that have acquired mutations that worsen antigen affinity, are eliminated through apoptosis. B cells with high affinity are positively selected through interaction with T follicular helper cells and ultimately differentiate into long-lived PCs, responsible for secreting antibodies, and MBCs, which keep memory of past infections and can rapidly respond to repeated antigen exposure.

The GC is histologically and functionally divided into two zones, the dark and the light zone. The dark zone contains rapidly proliferating B cells—cell cycles typically range between 6 and 12 h (3)—referred to as dark zone B cells or centroblasts (CBs). CBs undergo SHM, a process that randomly introduces point mutations with high frequency, ~ 10^−3^ per base pair per generation (5), into the IgV genes that encode for the antigen-binding site. CBs then migrate to the light zone of the GC and differentiate into light zone cells, or centrocytes (CCs), which are smaller, largely non-proliferative B cells that are committed to apoptosis unless rescued. CCs acquire antigen from follicular dendritic cells (FDCs) through their BCRs, ingest it and break it into peptides that are then exposed on the cell surface bound to the major histocompatibility complex II (MHC-II). The peptide–MCH-II complex is recognized by Tfh cells (6–8), which deliver survival signals and instruct the CC to either recirculate to the dark zone microenvironment to undergo additional rounds of hypermutation and selection, or promote GC exit and the differentiation into MBCs and PCs.

Earlier work by some of us produced a quantitative kinetic model of the GC that explained B cell differentiation into the PC compartment as a result of the interplay of a small module of three antagonistic transcription factors: B cell lymphoma 6 (BCL6), a potent transcriptional repressor required for the establishment and maintenance of GC; interferon regulatory factor 4 (IRF4) and B cell-induced maturation protein 1 (BLIMP), two essential regulators of PC development. In this model, these three genes were regulated by external cues mainly through the interplay between two signaling pathways, the BCR and CD40 signaling pathways (9). Newer experimental evidence has shown that the intensity of BCR signaling upon antigen binding controls the bimodal expression of IRF4, which in turn dictates B cell fate outcomes favoring the generation of PCs when antigens bind with high affinity (10, 11). In parallel, terminal differentiation of B cells into PCs has been described as a simple probabilistic process that is governed by a central gene-regulatory network and modified by environmental stimuli (12).

While we have a good comprehension about the molecular events underlying PC differentiation, the differentiation of GC B cells into MBCs is still incompletely understood. However, new experimental strategies developed over the last five years have enabled a more detailed study of MBC development, which has resulted in competing theories to explain the fate selection mechanisms of GC B cells (13–16). For instance, it was observed that GC B cells can divide asymmetrically, resulting in the differential segregation of key molecules within the cell, including, among other proteins, BCL6 (13). Asymmetric cell division can also result in unequal distribution of antigen among the cell progeny of the dividing CBs, and this polarized distribution can be maintained for extended periods of time (14).

Although asymmetric cell division generates an unequal inheritance of potentially fate-altering molecules in daughter cells, the functional importance of this observation for GC dynamics remains to be shown (12). Duffy et al. (15) produced an exhaustive study to determine whether the daughter cells of a dividing B cell exhibit asymmetric fates. Time lapse analysis of differentiation, death, and time to next division imaged from one cell division to the next, showed that daughters from B cell divisions largely undergo symmetrical fates. Interestingly, although a small proportion of B cell divisions displayed asymmetric cell fates—one daughter died and the other survived—the authors demonstrated that fate decision is largely determined by intracellular stochastic competition, i.e., independent but mutually exclusive processes working toward each fate, where the first process to be completed decides the cells' fate.

In parallel, Shinnakasu et al. (16) demonstrated that PCs have a significantly higher antigen affinity than MBCs, suggesting that antigen affinity (or a correlated quantity) plays a role in fate selection. Further evidence was provided by Ochai et al. (11), who demonstrated that increased antigen affinity favors generation of PCs. Furthermore, Weisel et al. (17) experimentally identified a temporal switch in the GC output, where MBC subsets with different immune effector functions are generated at earlier times, while long-lived, higher-affinity PCs are generated later in the GC response. It is worth noting that, whereas asymmetric cell division and stochastic competition are unable to explain a temporal switch in the GC without postulating unknown secondary mechanisms, a GC model based on affinity maturation can fit the observations: As GC B cells increase their antigen affinity during the lifespan of the GC, a higher PC output at later times is naturally expected if fate decision depends on antigen affinity.

In this paper, we expand previous models of the GC reaction (9) to account for the stochastic nature of cellular interactions within the GC with the goal of investigating several of the competing theories for B cell differentiation. Starting from the gene regulatory network controlling B cell fate decision established by some of the authors (9), we develop a comprehensive stochastic model of the main extracellular interactions taking place throughout the life of a GC B cell and use this model to explore the maturation and terminal differential of GC B cells.

## 2. Model

We present here a hybrid model of the GC that includes an intracellular molecular component, which accounts for regulatory events taking place during the terminal differentiation of B cells, and a stochastic extracellular model, which captures the cellular interactions and events taking place in the GC. Next, we briefly describe each model.

### 2.1. Intracellular model

Our starting point is an already published model of the GC that captures the transcriptional changes associated with the differentiation of GC B cells into PCs (9). The model is composed of a system of three differential equations describing the interplay between three transcription factors, BCL6, IRF4, and BLIMP, and two signaling pathways, BCR signaling and CD40 (see Supplementary Information for details). Dynamical analysis of the gene regulatory circuit reveals that non-linear effects dominate the transition from GC B cells into PCs. More specifically, a mathematical bifurcation on the levels of IRF4 generates a threshold below which only the states associated with GC cells are accessible. Above the threshold, the circuit irreversibly transitions to another state characterized by high levels of IRF4 and BLIMP1, corresponding to a terminally differentiated PC (18–21).

This early model did not consider the differentiation into MBCs. MBCs do not express BLIMP1 and have IRF4 expression levels above GC B cells, but below PCs (18). This implies that a bifurcation inducing permanently elevated IRF4 and BLIMP1 levels cannot have occurred in MBCs, and suggests a model where activated GC B cells with IRF4 levels above the threshold become PCs and cells below the threshold become MBCs. In addition, recent findings reveal that antigen intake by the BCR induces IRF4 (10, 11). Hence, we assume in our current model that when a CC is ready to leave the GC and differentiate, the amount of acquired antigen determines whether it becomes a PC or a MBC, in agreement with recent experimental observations (16).

To implement this, the system of ODEs previously defined (9) can be adapted by making IRF4 levels sensitive to the levels of antigen. Mathematical analysis reveals that the system dynamics is controlled by a dimensionless parameter β, roughly the ratio between all sources contributing to IRF4 induction vs. IRF4 degradation (see Supplementary Information for details):

(1) $$\beta =\frac{\text{IRF4 production}}{\text{IRF4 degradation}}\approx \frac{\mu_{r}+\alpha \text{antigen}+cd_{0}+\sigma_{r}}{\lambda_{r}*k_{r}},$$

where μ_*r*_, σ_*r*_, λ_*r*_, and *k*_*r*_ account, respectively for IRF4 basal transcription rate, induced transcription rate, degradation, and DNA dissociation constant. Their experimentally determined values are detailed in Table S1 in the Supplementary Information. In the above equation, α and *cd*_0_ are constants that account for the BCR and CD40 signaling-induced IRF4 transcription rate, and antigen stands for the amount of antigen a CC has acquired by the time it leaves the GC. To derive (Equation 1), we have assumed that CCs have already gone through an initial phase of BCR's induced BCL6 degradation, and hence, BCL6 is not actively repressing some of the genes associated with both signaling pathways (9). Our model also assumes that the amount of signaling delivered through the CD40 receptor is a digital on/off signal, and hence, CD40 signaling can be treated as a constant. Under these assumptions, all parameters in Equation (1) are constant except for the amount of acquired antigen, and hence, β depends linearly on the amount of antigen.

Equation (1) provides the mathematical basis to model B cell differentiation in our updated model. We assume that a CC can leave the GC only after it has received T cell help, and the value of β at that point determines whether it becomes a PC or MBC (see Supplementary Information). Using (Equation (1) and experimental data (17), we can find the threshold on β, antigen_threshold, above which a cell differentiates as a PC (Table 1). Hence, cells that leave the GC with antigen > antigen_threshold become PCs, otherwise, they differentiate as MBCs. This is in agreement with recent literature that has demonstrated that PCs have significantly higher antigen affinity than MBCs (11, 16).

**Table 1:** Parameter values and ranges of stability.

**Parameter**	**Lower bound**	**Fitted**	**Literature**	**Upper bound**
r_division_	0.10	0.11	0.11 (3, 22–26)	0.13
p_mutation_	0	0.4	0.39 (26, 27)	1
r_migration_	0.11	0.13	0.17 (28)	0.16
r_apoptosis_	0	0.21	0.10 (26)	0.74
r_FDC encounter_	0.004	0.035	0.021 (26, 29)	∞
r_T cell encounter_	0.26	1.92	0.07 (26, 27)	∞
r_activation_	0.19	1.20	2.00 (27)	26
r_recirculation_	0.54	1.08	0.49 (28)	∞
r_exit_	0	2.2	1.36s (26, 27)	4.0
antigen_threshold	0	0.22	–	∞

*The table reports a parameter set found by fitting the model to the GC output experimentally measured by Weisel et al. (17) (Fitted), and a second set estimated from parameters found in the literature (Literature). The Lower and Upper bounds columns report the lower and upper ranges of stability. Units: h^−1^, except p_mutation_ and antigen_threshold, which are unitless*.

While it might seem surprising that the dynamical output of our model is primarily governed by the kinetics of IRF4 (Equation 1), as demonstrated in the Supplementary Information, this is the result of mathematical inference, and not enforced by model construction. Our predictions reflect the central role of IRF4, whose graded response has been shown to be central to regulate the GC dynamics (10, 11, 30).

### 2.2. Extracellular Model

**Figure 1:**
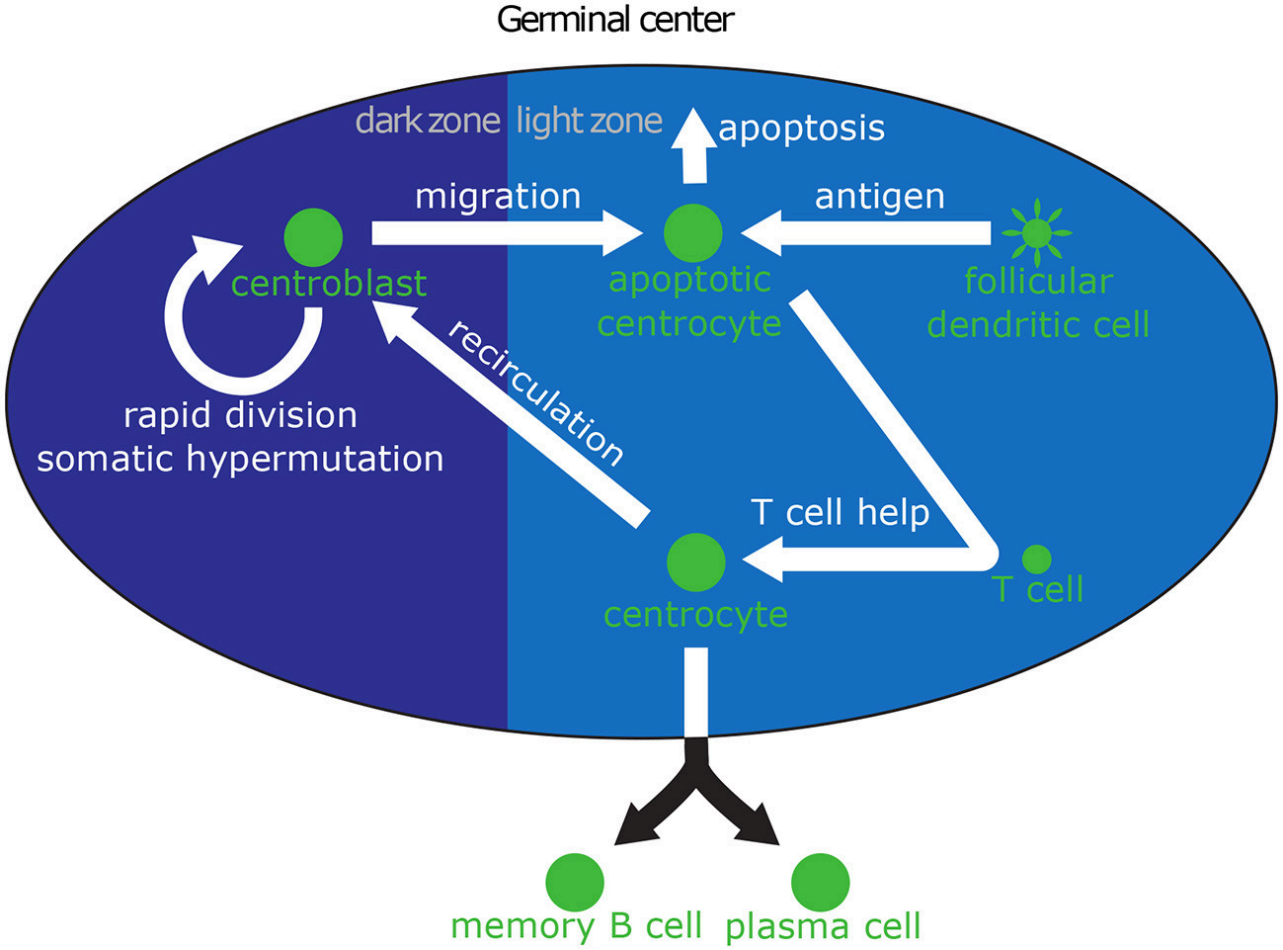
Biological interactions in the GC relevant to B cell differentiation. Centroblasts (CBs) can be seen on the left in the dark zone, where they rapidly divide and mutate their immunoglobulin V region. After a few hours, CBs migrate to the light zone and become centrocytes (CCs). High-affinity CCs can acquire large amounts of antigen from follicular dendritic cells (FDCs), which makes them more likely to receive survival signals from helper T cells. Low affinity CCs, which acquire little or no antigen, cannot compete for T cell help and die through apoptosis. CCs that have received survival signals may either recirculate into the dark zone for another round of division and hypermutation, or leave the GC and differentiate into PCs or MBCs.

The intracellular model determines the fate of a GC B cell as a function of the amount of antigen, which is acquired through interactions with FDCs. Hence, in order to accurately understand the GC dynamics, we need to model the extracellular interactions that take place through the life of a GC B cell and enable antigen acquisition. Minimally, the extracellular model should capture the most important molecular events that shape the life and fate of a B cell: SHM and affinity maturation as a CB in the dark zone; antigen acquisition and interaction with T cells as a CC in the light zone; recirculation into the dark zone for additional rounds of division and affinity maturation; and possibly death through apoptosis. The extracellular model is summarized in Figure 1.

#### 2.2.1. Stochastic Model

The events described in the extracellular component are stochastic in nature. For instance, the interaction with FDCs or T cells depends on the probability of encountering a FDC or a T cell, and BCR maturation depends on the random set of mutations that a CB acquires after each division. Hence, we model the extracellular component as a stochastic system composed of the following reaction channels:

(2) $$\text{CB}\overset{{\text{r}}_{\text{migration}}}{\to }{\text{CC}}_{\text{apoptotic}}\;,$$

(3) $${\text{CC}}_{\text{apoptotic}}\overset{{\text{r}}_{\text{apoptosis}}}{\to }\emptyset \;,$$

(4) $${\text{CC}}_{\text{apoptotic}}+\text{TC}\overset{{\text{r}}_{\text{T cell encounter}}}{\to }[\text{CCTC}]\;,$$

(5) $$[\text{CCTC}]\overset{{\text{r}}_{\text{activation}}}{\to }\text{CC}+\text{TC },$$

(6) $$\text{CC}\overset{{\text{r}}_{\text{recirculation}}}{\to }\text{CB },$$

(7) $$\text{CC}\overset{{\text{r}}_{\text{exit}}}{\to }\emptyset .$$

The probabilistic reactions (Equations 2–7) account respectively for the migration of B cells from the dark to the light zone, death by apoptosis in the light zone, encounter between a CC and T cell, CC activation by a T cell, recirculation to the dark zone, and differentiation. These reactions follow a simple mass-action kinetics, and hence can be simulated using the popular Gillespie algorithm (31), which generates statistically correct trajectories of a stochastic system of equations. However, the remaining reactions require more complex formulations, which we explain below.

#### 2.2.2. Centroblast Division

CBs in the dark zone divide according to the reaction channel:

(8) $$\text{CB}\overset{{\text{r}}_{\text{division}}}{\to }2\text{CB},$$

creating in the process two daughter cells, one of which randomly acquires mutations on its BCR genes through SHM. To model the random changes on the BCRs that occurred during division, we represent antibodies in a 4D shape space where each antibody shape, Φ_antibody_, is mapped to a point (32). Mutations occur according to a given mutation probability, p_mutation_, and are modeled by a jump to a neighboring position in the shape space (in an arbitrary direction). After a mutation takes place, the affinity between the mutated antibody and the antigen is computed as the Hamming distance between the antibody's new position and the origin ||Φ_antibody_−Φ_origin_||_H_, which represents the clone of maximum affinity to the antigen (26, 27). The binding probability, given by the antigen–antibody affinity, is calculated from a Gaussian distribution with width σ = 2.8 (27, 33), i.e., $\text{affinity}\propto \exp\left(-\frac{||\Phi_{\text{antibody}}-\Phi_{\text{origin}}|{|}_{\text{H}}}{2.8^{2}}\right).$ Regarding antigen, any amount acquired from previous interactions with FDCs is divided equally among the daughter cells. We examine later on in this paper an alternative scenario, where one daughter cell inherits all antigen (see “*Asymmetric cell division”* discussion in section 4).

#### 2.2.3. Antigen Uptake

CCs that encounter FDCs might acquire antigen if their BCRs bind with enough affinity to the antigen. Our model assumes that all FDCs carry the same amount of antigen, which is exposed on their surface. We assume that antigen can only be acquired from the FDCs and the amount presented reflects the concentration of antigen complexes in the extracellular milieu (3). Our model does not explicitly simulate FDC dynamics, but considers that antigen uptake occurs when a CC encounters an FDC through the following reaction channel:

(9) $${\text{CC}}_{\text{apoptotic}}\overset{{\text{r}}_{\text{FDC encounter}}}{\to }{\text{CC}}_{\text{apoptotic}},$$

upon which, the CC acquires antigen in an affinity-dependent manner:

(10) $${\text{antigen}}_{\text{new}}={\text{antigen}}_{\text{old}}+a_{0}*\text{affinity}.$$

Notice that in our model, the amount of acquired antigen is a relative quantity that is only used to rank CCs when competing for T cell help, and hence we have a degree of freedom that we use to set the constant *a*_0_ = 1.

#### 2.2.4. Centrocyte Substitution

When a CC encounters a T cell, the CD40 receptor of the CC binds the CD40L ligand on the surface of the T cell. The T cell delivers signals necessary for CC activation. This process is simulated as an apoptotic CC and a T cell forming a compound [CCTC], which dissolves into a non-apoptotic CC and a T cell at a rate *r*_activation_. Competition between CCs for this T cell help is simulated using a substitution reaction: If a second CC encounters the CC and T cell compound, the CC with the lower antigen amount returns to the apoptotic state.

(11) $$\left[{\text{CC}}_{1}\text{TC}]+{\text{CC}}_{2,\text{apoptotic}}\overset{{\text{r}}_{\text{T cell encounter}}}{\to }{\text{CC}}_{1,\text{apototic}}+[{\text{CC}}_{2}\right]$$

Importantly, the reaction in Equation (11) can only occur if antigen(CC_2_) > antigen(CC_1_). According to Equation (11), two CCs can simultaneously interact with the same T cell, similarly to previously published models (27). Once a CC and T unbind, they are both released and can further interact with other T cells and CCs respectively.

Simulation of GC B cells requires keeping track of the BCR shape space position and the amount of antigen a cell has acquired. Moreover, the centrocyte substitution reaction introduces direct competition between CCs, where small differences in antigen are relevant for an accurate simulation. This requires an extension to the Gillespie algorithm to allow for the efficient simulation of particles with continuous properties described in the next section.

## 3. Algorithm Description

The Gillespie algorithm (31) is commonly used to simulate trajectories of stochastic systems. A trajectory of a single simulation represents a sample from the probability mass function, which is the exact solution of the master equation. The Gillespie algorithm is frequently used to simulate biochemical systems; however, its direct application to our GC model presents some difficulties.

The Gillespie algorithm consists of discrete reaction steps, during which a single reaction is selected according to the propensities, or instantaneous probabilities. The propensities are computed according to mass action kinetics, i.e., as the rate constant of each reaction multiplied by the abundance of reactants. Since all propensities must be calculated to perform one step, each step has the computational complexity O(N_reactions_). Furthermore, the standard Gillespie algorithm assumes that all particles in a given species are identical. In our GC model, however, CBs and CCs need to keep track of two individual properties: the amount of acquired antigen and the shape of their BCR. A straightforward way of modeling these continuous properties in the Gillespie framework would be to discretize them into bins of discrete property values. Note that the desired accuracy of the approximation increases quadratically with the number of bins (and hence reaction channels). Similarly, the complexity and computational time also grows quadratically with the number of bins, as the propensities of each reaction channel have to be calculated at each simulation step, even though the state might not be populated at the time. We note that, as both properties play a fundamental role in explaining the GC dynamics, reducing the number of discretization bins to speed up the simulation can lead to erroneous predictions, e.g., a coarse antigen discretization might result in a large number of ties in the CC substitution reaction (see Equation 11).

To avoid expensive calculations or poor precision due to coarse discretization, we extend the Gillespie algorithm to enable the efficient tracking of individual cells and properties. Before outlining our algorithm, we notice that the two cellular properties—antigen and BCR shape—affect only the substitution reaction channel, Equation (11), and therefore, all other reactions can be modeled with the standard Gillespie algorithm. Regarding the CC substitution reaction, an unbound centrocyte, CC_2_, can replace a centrocyte bound to a T cell, CC_1_, if and only if CC_2_ has captured more antigen than CC_1_. Our algorithm proceeds in two steps. First, it assumes that all CCs are identical and draws CC_2_ according to a uniform distribution from the available pool of unbound cells. Second, it compares the amount of antigen acquired by CC_1_ and CC_2_ and rejects the reaction if antigen_2_ < antigen_1_. This approach is an example of Poisson thinning, a process whereby propensities that include undesired reactions are computed and subsequently rejected, and is mathematically consistent with the Gillespie algorithm (34–36). A pseudo-code description of our algorithm is given in Algorithm 1, and a detailed explanatory example of the modified algorithm can be found in the Supplementary Information.

**Algorithm 1:**
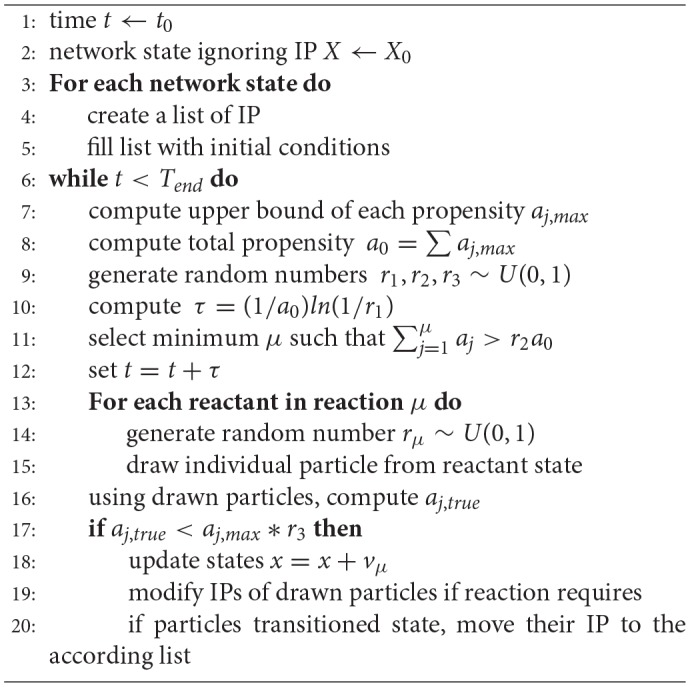
Stochastic Simulation Algorithm with individual properties (IP)

### 3.1. Comparison to the Standard Gillespie Algorithm

A faithful Gillespie algorithm requires all individual particles of a species to be identical. Therefore, individual properties must be expressed as discrete separate species. If high precision is needed or multiple properties must be discretized, the number of discretization buckets might become very large, which results in a high computational cost. Aside from the computation burden, such algorithm would also become inefficient, as the propensities of many empty reaction channels would need to be computed at each step, independently of whether the state is populated or not. On the other hand, discretization using a low number of buckets leads to low precision and might not faithfully recapitulate the biological evolution of the system in cases where there is competition between particles based on the value of a property, as in the CC substitution reaction (11).

In comparison, our algorithm offers machine precision and a much lower computational cost (since no unnecessary propensities need to be computed), at the cost of a larger memory footprint due to the lists of property values that need to be kept for all species with properties. While we have not experienced any loss of performance in our simulations, in theory, if the number of particles is very high, the algorithm could become memory-bounded. Also, our algorithm achieves faster simulation times by computing more reaction steps (associated with the rejected reactions). Also theoretically, if *r*_max_ >> *r*_average_, the algorithm might compute mainly rejected reactions, decreasing performance. However, such unfavorable scenario could be mitigated by using other simulation approaches, such as tau-leaping, where reactions are computed for an interval of length tau before updating the propensity functions (37). Finally, our algorithm requires an increase in computations per reaction due to the drawing of individual properties and updates of particles counts once a reaction channel associated with a property has been selected (see Algorithm 1 and Supplementary Information for details).

Despite the drawbacks, our algorithm offers machine precision and fast simulation times for stochastic systems with individual particle properties. Therefore, it enables the simulation of systems with individual particle properties whose complexity or number of dimensions made a Gillespie simulation previously unfeasible. If the system does not suffer from very large *N* or *r*_max_ >> *r*_average_, this algorithm even offers a performance increase over a faithful Gillespie algorithm because no additional propensities need to be computed.

### 3.2. Algorithm Performance

To compare the performance of Algorithm 1 to that of the traditional Gillespie algorithm, we evaluated their running times simulating a toy system, which we describe in the SI section “*Gillespie algorithm with continuous particle properties*.” Using both the traditional and our extended Gillespie algorithm, we simulated our toy system using an increasing number of discretization bins, corresponding to higher simulation precision (see Figure S1, left subplot). The Gillespie algorithm only outperforms our algorithm at very low precision levels (below 10 bins). In the right subplot in Figure S1, we evaluate the running times of both algorithms, where traditional Gillespie uses 10 bins, a regime where both simulation approaches show similar performance. The figure shows that both algorithms scale linearly with the number of particles in the simulation, i.e. the increased memory footprint did not affect the performance.

### 3.3. Model Parameters and Initial Conditions

We follow two different approaches to determine model parameters. First, we compute estimates for each parameter using published literature and experiments (see details in the Supplementary Information). Second, we fit parameters to reproduce experimental data of the GC output over time, such as those reported by Weisel et. al (17). Both sets of parameters are summarized in Table 1.

Regarding initial conditions, our simulations start with 100 CBs, 10 FDCs, and 10 T cells (Table 2). Assuming a doubling time of ~10 h for CBs (3) and starting from one founding activated B cell, a population of 100 CBs can be reached in ~66 h [= 10 h * log(100)/log(2)]. Thus, our simulations start during the active phase of clonal expansion, while the first CBs are about to migrate to the light zone.

**Table 2:** Initial conditions used in model simulations.

	**CBs**	**CCs**	**TCs**	**FDCs**
Initial counts	100	100	10	10

**Figure 2:**
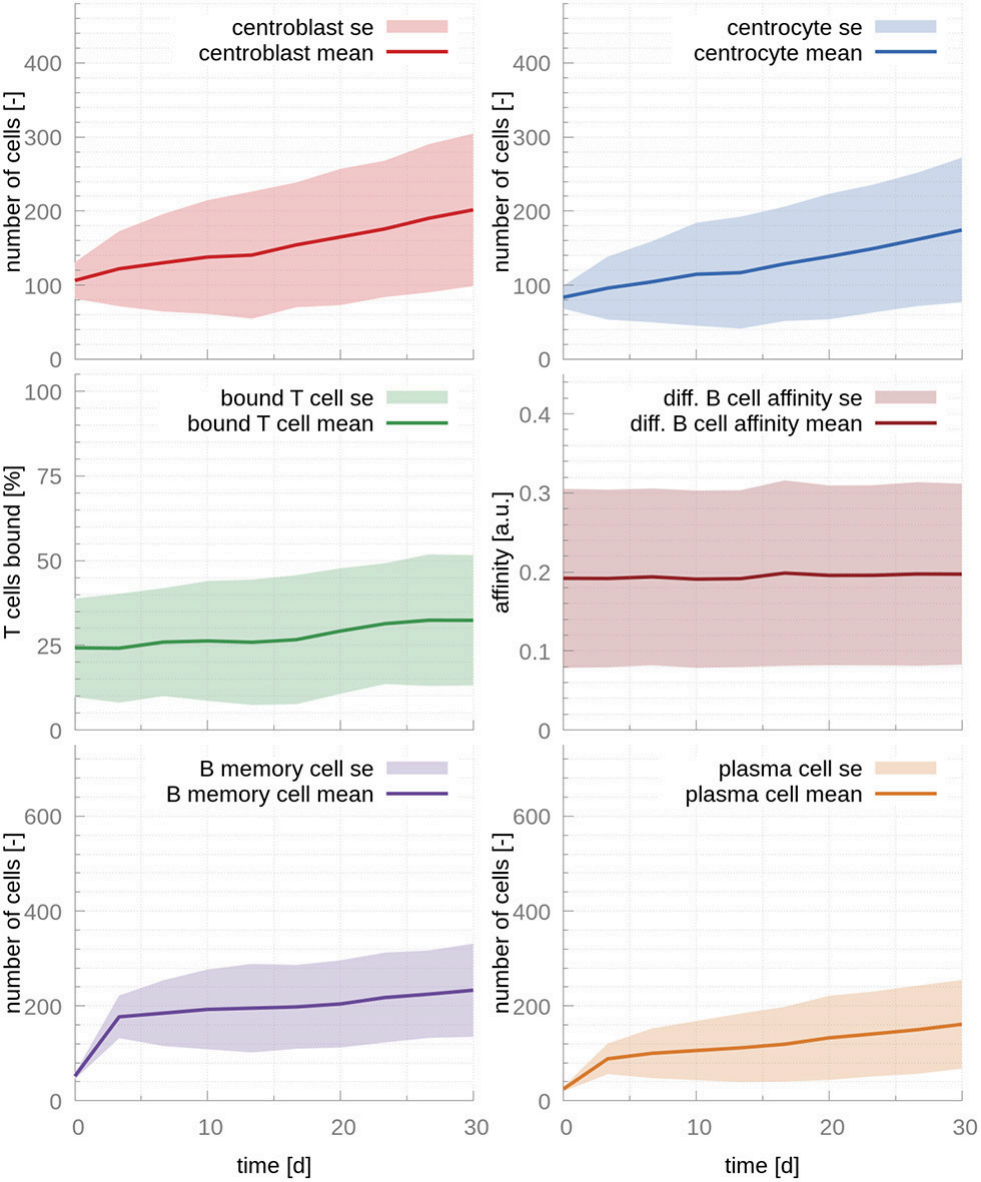
GC cellular populations over time for the set of stabilized literature parameters. The parameters calculated from evidence in the literature, adjusted to lead to stable populations. Affinity does not increase over time, MBC output is still significant at day 30 and PC output only reaches steady state at late time points, contrarily to experimental evidence (17). The ratio of T cells interacting with B cells never exceeds 35%, which results in insufficient competition for T cell help. In the figures above, germinal center cell counts are the average number of concurrent cells, while cells leaving the germinal center are accumulated per periods of 18 h. The shaded area depicts standard error (SE).

**Figure 3:**
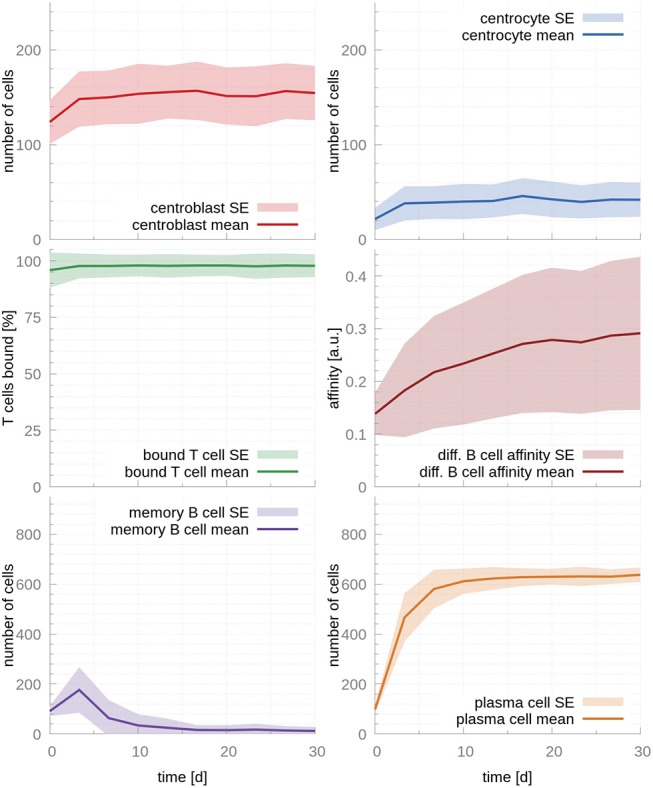
GC cellular populations over time for the set of fitted parameters. Parameters were fitted to experimental observations of the GC output over time (17). Faster affinity maturation leads to a transient output of MBC at early time points that dwindles and vanishes by day 10. In parallel, PCs reach a strong and steady output by the time of the temporal switch (day 10). During the whole life of the GC, the majority of T cells are bound, enabling competition between CCs and, hence, production of PCs. In the figures above, germinal center cell counts are the average number of concurrent cells, while cells leaving the germinal center are accumulated per periods of 18 h. The shaded area depicts standard error (SE).

**Figure 4:**
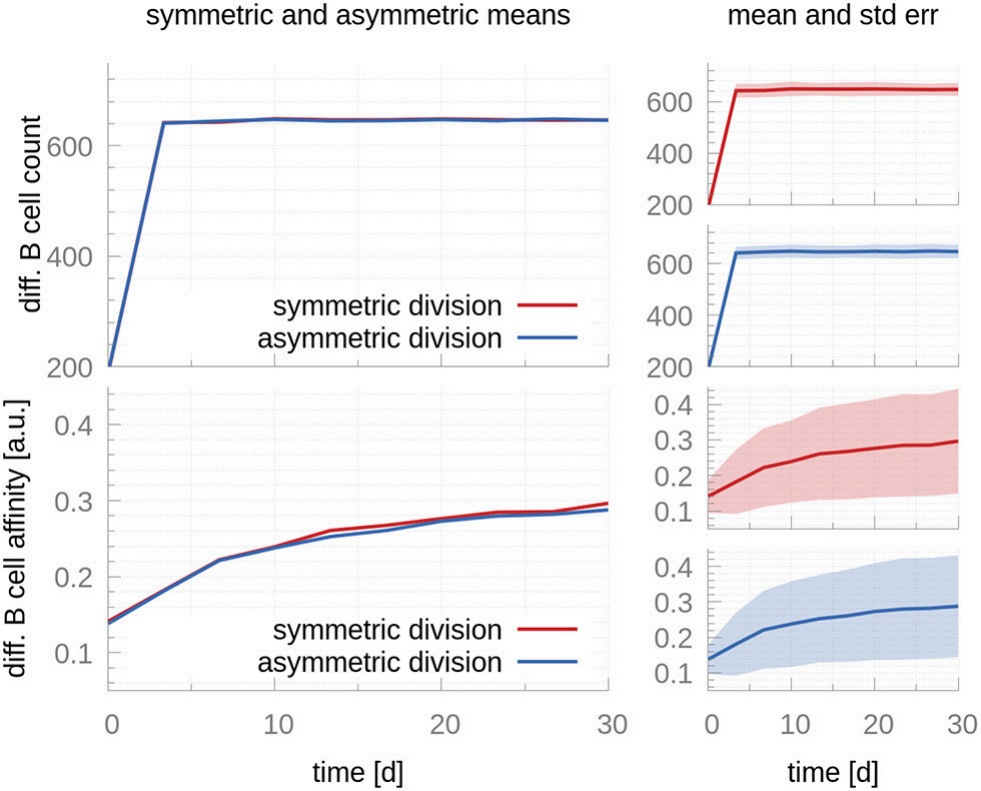
A comparison between symmetric and fully asymmetric distribution of antigen during centroblast division. Top row shows the mean **(Left)** and mean plus standard error **(Right)** of the total differentiated GC output in the symmetric and asymmetric scenarios. In the former, 50% of the antigen goes to each CB daughter cell after each division; in the latter, 100% of the antigen goes to a single daughter cell. The bottom row reports the affinity of the differentiated GC cells. Differences between both scenarios are not significant both in terms of the total cell counts and the affinity of differentiated B cells. Since the number of differentiated cells is a discrete number, its standard deviation cannot be evaluated by taking a snapshot at a specific time. Instead, we accumulate the cells leaving within 3-day-intervals, as in the experimental data we fit the model to Weisel et al. (17). These intervals cause the sharp turn at day 3.

*We assume that our simulations start at an early stage of the GC development, after a phase of monoclonal expansion. As is evident from the different simulations (Figures 2–4), after an initial transient period lasting < 10 days, all simulations converge to steady-state values. The number of FDCs and T cells are taken from Meyer-Hermann et al. (26)*.

### 3.4. Numerical Stability Analysis

In order to understand the ranges of parameters where the system shows a stable behavior, a stability analysis was performed. Due to the combinatorial explosion intrinsic to models with even a moderate number of parameters, a global stability analysis where we examine the effect of simultaneous changes in all parameters is cost prohibitive. Instead, we perform a local stability analysis, where we vary each parameter independently while keeping the other fitted parameters constant. Due to the stochastic nature of the simulations, it is not immediately obvious whether a set of parameters is stable or unstable. We therefore define that a single simulation has diverged if the number of cells in the GC is outside the interval of [1, 5000] cells. If more than 100 out of 200 simulations diverge, we consider the parameter set to be unstable. To confirm that our conclusions do not depend on the chosen size limit of the GC, in our case 5000 cells, we also repeated the analysis with a much higher GC size of 25,000 cells, and found negligible differences. We note that in our model, the size of the GC is a computational prediction derived for a set of parameters, and not an externally imposed parameter itself. The upper limits on the GC size used for the numerical stability analysis were chosen according to the typical GC sizes found in our simulations and only used to determine parameter stability bounds. A lack of experimental evidence for the antigen threshold that selects cells to the MBC or PC pools prevents us from deriving its value from published articles. Instead, we used the fitted value in all simulations. Table 1 reports the stable range for each parameter.

### 3.5. Parameter Optimization

One of the parameter sets in Table 1 was determined by fitting the model to experimental data (17). The reported parameters are the ones that minimize the following criterion:

(12) $$l=\sum_{i}|\theta_{m,i}-\theta_{e,i}|+|\theta_{m,i}^{2}-\theta_{e,i}^{2}|\;,\;i=\{1\;(\text{PM}),2\;(\text{MBC})\},$$

where θ_*e*_ are the experimentally determined normalized counts of PCs and MBCs that exit the GC over a period of 30 days, as measured by Weisel et al. (17), and θ_*m*_ are the respective model predictions. The criterion defined by Equation (12) aims to minimize differences in means and standard deviations between experimentally measured and computed counts. The optimization was performed using maxLIPO from dlib (38).

## 4. Results

### 4.1. T Cell Help Is Crucial for Affinity Maturation and PC Production

Stochastic simulations with the parameters found in the literature proved to be unstable, with all populations vanishing by day 10 (see Figure S2). A deterministic analysis (see SI) revealed that the ratio $\frac{{\text{r}}_{\text{division}}}{{\text{r}}_{\text{migration}}}$ tightly controls the regime of stability. A numerical stochastic exploration of the stability bounds of the fitted parameters revealed the following condition for a stable regime: $0.72<\frac{{\text{r}}_{\text{division}}}{{\text{r}}_{\text{migration}}}<0.98.$ Inserting the parameters into the constraints found in the deterministic analysis yielded the same bounds within a deviation of 1%. These bounds explain why the set of parameters derived from the literature did not lead to stable populations: The parameters found in the literature result in a ratio of $\frac{{\text{r}}_{\text{division}}}{{\text{r}}_{\text{migration}}}=0.65$, clearly outside the stable bounds.

To confirm that these two parameters are causing the unstable behavior, we modified r_migration_ in order to bring the system back into the stability window. A change from 0.17 to 0.12 results in stable populations that, however, do not capture the trends observed in physiological GCs, e.g., affinity maturation with time and a temporal switch from MBC production to PC production (Figure 2). Further investigation reveals that in this dynamical regime many T cells are free and rarely bind CCs, which leads to inefficient competition for T cell help. Indeed, for this set of parameters about 70% of T cells are unbound at all times (Figure 2). Another way of understanding why there is no affinity maturation in this system is by considering the T cell encounter rate. In the set of parameters derived from the literature, this rate is 0.07*h*^−1^, resulting in CCs needing ~14.3*h* on average to encounter a T cell. This large waiting time is higher than the mean life-time of a CC before it dies through apoptosis, which has been estimated to be ~10*h* (27). Hence, for these parameters, an average CC does not have enough time to find a T cell and efficiently compete for survival signals. To demonstrate the importance of allowing for enough time for CCs to encounter and interact with T cells, we performed an additional simulation where we increased three-fold r_T cell encounter_ (see Figure S3). As it is evident in this figure, the fraction of bounded T cells increases to 80 %, leading to a system that exhibits affinity maturation with time. However, affinity maturation is slow, resulting in a noticeable output of MBCs at late time points and a slow increase of the PC output with time, which only starts reaching steady state at day 30, in disagreement with experimental observations (17).

Our findings regarding the importance of competition for T cell help is in agreement with previous mathematical models (26) and experimental evidence (28) that demonstrated that T cell help is the limiting factor in GC selection.

### 4.2. Affinity Maturation Can Explain the Temporal Switch in the GC Output

Next, we fit the model parameters to data kindly provided by Weisel et al. (17), who experimentally determined the amount of MBCs and PCs leaving the GC over its lifetime. Fitted parameters are reported in Table 1. As expected, the fitted T cell encounter rate is much higher, r_T cell encounter_ = 1.9*h*^−1^, leading to a faster increase of affinity over time (Figure 3). Importantly, this affinity maturation results in a significant time shift in cell output, where MBCs are only produced at early times (production peaks at day 3) and PCs become the dominant cellular output of the GC afterwards, in agreement with experimental observations (17). Supporting our previous observation that T cell competition is critical for PC production, 97% of T cells are bound after the system reaches steady state, occurring between the 3rd and the 10th day after the establishment of the GC.

### 4.3. Asymmetric Cell Division

One theory that has gained attention to explain the B cell fate decision between MBC and PC is asymmetrical cell division, where the different fate between GC B cells is explained as a result of unequal inheritance of key molecular determinants, among which are the GC master regulator BCL6 and antigen (13, 14). To test this theory, we examined the impact of asymmetric distributions of the concentration of key transcription factors and antigen using our model.

Fist, concerning a possible unequal distribution of key transcription factors, in the absence of a mechanism that can maintain the out-of-homeostasis levels, the concentration of a transcription factor will return to its initial value after a certain relaxation time. In order for the asymmetric division to have an influence on B cell differentiation, such relaxation time must be longer than the time between the CB division and B cell differentiation. The relaxation time can be estimated to be τ_relaxation_ = 0.46 h (see Supplementary Information for details). Similarly, we can estimate the average time from a CB division to B cell differentiation to be τ_differentiation_ = 9.49 h (Supplementary Information). Considering these numbers, it seems unlikely that asymmetric distribution of transcription factors during CB division can influence the cell fate decision in the absence of additional mechanisms to maintain the asymmetry. To our knowledge, no such mechanism has been reported in the GC. Therefore, the current model does not support a significant role for asymmetric cell division in fate decision.

Antigen has also been shown to be inherited in an asymmetric manner, and this asymmetry has been shown to have implications for the progeny fate: Daughter cells receiving larger antigen stores exhibit a prolonged capacity to present antigen, which renders them more effective to compete for T cell help (6, 14). On the other hand, daughter cells receiving smaller amounts of antigen might receive less or no T cell help, compromising their chances to leave the GC and differentiate. We use our model to investigate the effects of asymmetric antigen distributions following CB division. Even though previous authors used more similar distributions and lower frequencies (14), we consider the most extreme scenario: fully asymmetric antigen distribution in all CB divisions, meaning all antigen is inherited by only one CB daughter cell in all divisions. Figure 4 compares the output of the asymmetric and symmetric models. No changes in affinity maturation or cell population dynamics are noticeable, suggesting the distribution of antigen in the daughter cells following a CB division does not play a significant role in the processes associated with affinity maturation or PC production.

## 5. Discussion

We have presented here a hybrid model of the GC that explains the terminal differentiation of GC B cells as a result of the interplay between intracellular genetic regulation and stochastic extracellular events. To efficiently simulate our model, we have developed an extension of the Gillespie algorithm that enables the simulation of stochastic systems with continuous particle properties without incurring high computational costs or poor precision due to discretization. We expect that this extension might find application to other stochastic problems where discretization is necessary.

Our model was able to recreate the temporal switch in GC output from MBCs to PCs by merely assuming that B cell fate decision is driven by the amount of antigen uptake. A deterministic stability analysis revealed the existence of a stability regime controlled by the ratio between the rate of CB division and CB migration to the light zone to become a CC. We interpret these bounds as the need for tight regulation on CB division and migration: too much division and little migration lead to fast and uncontrolled growth of the CB population; too little division and high rates of migration lead to the rapid dissolution of the GC, as differentiated or apoptotic cells are not replaced rapidly enough.

Another important prediction of our intracellular model is the central role of IRF4, whole levels play a major role in determining B cell fate. This is in agreement with experimental evidence that have shown that IRF4 is expressed in a graded manner in differentiating B cells, with high concentrations inducing BLIMP1 and plasma cell differentiation (30). Subsequent work has demonstrated that the intensity of signaling through the BCR controls the bimodal expression of IRF4, which dictates B cell fate outcomes (10), and a *kinetic control* model based on IRF4 has been postulated (11). It is likely that intermediate levels of IRF4, which in our models are associated with MBC differentiation, correlate with the activity of additional unknown transcription factors that drive the differentiation. Further experimental characterization of these factors will enable the improvement of any GC quantitative model.

In that respect, the intracellular model that we introduce in the Supplementary Information is a minimal transcriptional model that aims to explain the terminal differentiation of B cells, and as such, it is only based on 3 transcription factors, BCL6, IRF4 and BLIMP1, all of them master regulators of the GC and B cell differentiation. Our model is not meant to be complete, and indeed, there is increasing experimental evidence of additional factors that might play a role in regulating MBC differentiation (39, 40). For instance, at the transcriptional level, BACH2 has been shown to be highly expressed in the GC B cells prone to enter the memory pool (16). Furthermore, BACH2 has also been reported to co-bind to the DNA together with BCL6 to repress BLIMP transcription (41). However, mathematical analysis of an extended model including BACH2 showed no additional predictive benefit, as its activity strongly correlates with BCL6 activity (See Supplementary Information). Hence, we opted for a minimal transcriptional model to reduce unnecessary complexity that could hinder our models inference power. In doing so, we have taken a similar approach to other modeling efforts that have treated BCL6 and BACH2 as a single species (10).

To simulate our model, we derived two sets of parameters, one extracted from the literature and a second one fitted to reproduce experiments of the GC output (17). Inspection of the simulation predictions of both sets of parameters showed that the parameters derived from the literature fell outside a narrow window of stability, leading to unstable populations with early decay. On the other hand, the set of fitted parameters produced a realistic scenario with noticeable affinity increase over time and a temporal switch from MBC to PC production by day 10. Simulations on both sets of parameters demonstrated that competition for T cell help is critical to achieve efficient GC cellular output. For instance, the fitted parameters have a rate of encounter between T cells and CCs that is ≈ 27 times larger than the rate derived from the literature, resulting in most T cells being bound at all times, and hence enabling efficient competition for survival signals.

The extracellular model is probabilistic in nature, meaning that cells with the same affinity might have different outcomes, although with different probability. For instance, an intermediate affinity cell might leave the GC as a MBC, but it also has a non-zero probability of staying and increasing its affinity, potentially differentiating as a PC. This probabilistic nature results in a continuous output of differentiated B cells in agreement with experimental evidence, which have shown that PCs and MBCs leave the GC throughout the response (17, 42).

Furthermore, we showed that asymmetric cell division is unlikely to cause fate selection in the absence of additional external factors that can maintain a polarized distribution of key molecular players. For instance, our analysis demonstrates that asymmetric distribution of key transcription factors does not persist until the differentiation time. Indeed, the time elapsed from the moment a CB divides for the last time until it can leave the GC, which we calculated to be ~9h, defines a threshold of *GC memory*: Any perturbation that can be erased in shorter times will not have any effect on the GC average output. These considerations apply to any molecular entity that can be asymmetrically distributed after a CB division. We also explore computationally the impact of asymmetric inheritance of antigen and show that it does not lead to any noticeable difference with respect to a symmetric antigen distribution model. Of course, we cannot rule out the possibility that additional factors not characterized yet might be at play during asymmetric cell division, resulting in cell fate regulation.

Our model represents a first step toward the multi-scale characterization of the GC and aims to capture the events that underlie the temporal switch in the GC output from MBCs to PCs. Several extensions are possible. For instance, T cells are considered fixed entities external to our model, and hence, their number is constant. However, experimental evidence shows that T cells can migrate to different GCs and newly activated T cells can invade preexisting GCs (43). A possible extension of our framework would be to consider T cells as stochastic particles whose number is governed by biological principles, modeling hence the observed dynamical communication between GCs.

Regarding the interaction between CCs and T cells in the light zone, it has been recently proposed that T-B cell interaction might happen as an entanglement, a form of contact that involves more extensive surface engagement and thus more efficient delivery of signals (44). While a detailed model of an entangled state between CCs and T cells would require the implementation of molecular dynamic techniques, which are beyond the scope of this paper, we have taken one step in the direction of building more realistic models of the GC reaction by explicitly accounting for the signaling and transcriptional events taking place inside CCs following interaction with T cells.

We also note that our model assumes a binding time of 30 min before a CC becomes activated and can differentiate (27). This is an approximation of the T–B cell interaction patterns reported *in vivo*, where B cells integrate signals from many short contacts of approximative 5 minutes with T cells (22), and the amount of integrated signal determines whether a B cell recirculates to the dark zone or differentiates as either a memory B cells or plasma cell. In our model, however, these short contacts associated with a time scale of a few minutes are very unlikely to produce an imprint in the GC output in normal physiological conditions lasting several weeks. Similarly to other well-studied systems with very different time scales, such as Michaelis–Menten kinetics, the shortest time scale (the binding-unbinding of the enzyme and substrate in the Michaelis–Menten example) can be assumed to reach fast quasi-equilibrium and its dynamics disregarded in a first approximation.

Finally, our model is not aimed to capture early or late events that might underlie the establishment and shutdown of the GCs. For instance, our model does not account for an initial phase of monoclonal expansion, which takes place from day 3 to 7 in the dark zone. This phase, if included in the model, might have the effect of delaying our simulations by a few days and increasing the number of GC B cells, resulting in better agreement with experimental data (17). Similarly, antibody-feedback has been proposed as a mechanisms by which GC B cells govern their own fate (45). Specifically, antibodies secreted by GC B cells can limit antigen access and influence B cell selection, plasma cell output, T cell interaction, as well as terminate the GC reaction. Although our model does not capture these events, we do not expect that their modeling will change the main conclusions of this paper, namely, the importance of T cell competition and the role of antigen affinity in determining B-cell fate.

To summarize, we have presented here a multi-scale hybrid model of the GC consisting of intracellular and extracellular components that enables the investigation of current theories regarding the maturation and differentiation of GC B cells. The model explains the shift from MBC to PC production over the lifetime of the GC and presents evidence linking MBC fate selection to antigen affinity maturation. Understanding the dynamics of the GC as well as the terminal differentiation of B cells is key to understanding the dynamics of the adaptive immune system. Our model allows the exploitation of new experimental evidence and the testing of theories about B cell maturation and differentiation.

## Data Availability

The datasets for this manuscript are not publicly available because we used data from Weisel et al. (17). Data was available only upon request. Requests to access the datasets should be directed to Weisel et al. (17).

## Author Contributions

JL, MRM, and MT conceived the study and analyses. MT implemented the framework and performed data analysis. UK provided biological analysis and interpretation. MT and MRM wrote the manuscript with input from all authors.

### Conflict of Interest Statement

MT and MRM were employed by IBM while this work was conducted. The remaining authors declare that the research was conducted in the absence of any commercial or financial relationships that could be construed as a potential conflict of interest.
