# Supplementary material for: A Probabilistic Model of the Germinal Center Reaction
Source: Front Immunol. 2019 Apr 3;10:689. doi: 10.3389/fimmu.2019.00689 (PMC6456718; doi:10.3389/fimmu.2019.00689)
Supplement: Supplementary file 1 [file Data_Sheet_1.pdf]

Supporting Information of "A probabilistic model of the germinal center reaction"

Marcel Jan Thomas[a,b], Ulf Klein[c], John Lygeros[b], and María Rodríguez Martínez[a]

[a]IBM Research Zürich, Säumerstrasse 4, 8803 Rüschlikon, Switzerland
[b]ETH Zürich, Automatic Control Laboratory, Physikstrasse 3, 8092 Zürich, Switzerland
[c]Experimental Haematology, Leeds Institute of Medical Research at St. James's, University of Leeds LS9 7TF, UK

Intracellular model of the germinal center reaction

Earlier work by some of the authors produced a quantitative kinetic model of the germinal Center (GC) that explained B cell differentiation into plasma cells (PCs)[1]. The model uses ordinary differential equations to capture the interplay of a small module of three antagonistic transcription factors:

- **BCL6**: is considered a master regulator of the GC reaction. It is expressed in most GC B cells[2–4] and necessary for the GC formation[2,5,6]. Among its regulatory functions, BCL6 represses DNA damage repairs[3,7,8], which would hinder somatic hypermutation (SHM) and transcriptionally represses BLIMP1[7,9–11].
- **BLIMP1**: is considered a master regulator of the PC phenotype[7]. Highly expressed in PCs [7,12,13], it induces plasmablast-like features in centrocyte (CC) cells[13] and is required for PCs to form[14]. Furthermore, it transcriptionally downregulates BCL6[7].
- **IRF4**: upregulates BLIMP1 and has therefore been theorized to effect PC differentiation[7].

In addition, the original model included two signaling pathways, B cell receptor (BCR) and CD40, that transduce external signals respectively delivered by follicular dendritic cells (FDCs) and helper T cells. Namely, FDCs expose antigen that binds to the BCRs on B cells and initiates a signaling cascade that results in the inhibition of BCL6 and the induction of IRF4. Helper T cells expose CD40L, a ligand present on the surface of helper T cells, that binds to the CD40 receptor and leads to B cell activation. Dynamical analysis of the model revealed the existence of two stable steady states representing CCs – high BCL6, low IRF4 and low BLIMP1 – and PCs – low BCL6, high IRF4 and high BLIMP1.

According to the original model, BCR stimulation inhibits BCL6, while CD40 induces IRF4. However, more recent findings support the idea that BCR stimulation, which depends on antigen uptake, may also induce IRF4 [15,16]. Hence, our current model reflects this newer understanding:

$$\frac{dp}{dt} = \mu_p + \sigma_p \frac{k_b^2}{k_b^2 + b^2} + \sigma_p \frac{r^2}{k_r^2 + r^2} - \lambda_p p \,, \tag{S1}$$

$$\frac{db}{dt} = \mu_b + \sigma_b \frac{k_p^2}{k_p^2 + p^2} \frac{k_b^2}{k_b^2 + b^2} \frac{k_r^2}{k_r^2 + r^2} - (\lambda_b + bcr)\, b \,, \tag{S2}$$

$$\frac{dr}{dt} = \mu_r + \sigma_r \frac{r^2}{k_r^2 + r^2} + bcr + cd40 - \lambda_r r \,, \tag{S3}$$

where p, b and r stand for the protein levels of BLIMP1, BCL6 and IRF4 respectively; the μ's represent the basal production rate of each protein; the λ's stand for the degradation rates; σ represents induced transcription rate; and k are the dissociation constants, i.e. the ligand concentration that produces half of the maximum induced transcription rate. The model implicitly assumes that the protein levels are proportional to mRNA levels. bcr and $cd40$ denote the respectively intensity of signaling through the BCR and CD40 pathways. As BCL6 represses some of the genes associated with both signaling cascades, the following phenomenological forms are assumed:

$$bcr = \alpha \, \text{antigen} \cdot \frac{k_b^2}{k_b^2 + b^2} \,, \tag{S4}$$

$$cd40 = cd_0 \cdot \frac{k_b^2}{k_b^2 + b^2} \,, \tag{S5}$$

where α and cd_0 are constants and we assume that the intensity of BCR signaling increases linearly with the amount of acquired antigen[15,16] is linear.

Mathematical analysis reveals that, after an initial phase of BCR-induced BCL6 degradation and once the regime $k_b \gg b$ has been reached, the system dynamics is controlled by the dimensionless parameter β, defined as:

$$\beta = \frac{\text{IRF4 production}}{\text{IRF4 degradation}} \approx \frac{\mu_r + bcr + cd40 + \sigma_r}{\lambda_r * k_r} \tag{S6}$$

$$\approx \frac{\mu_r + \alpha\,\text{antigen} + cd_0 + \sigma_r}{\lambda_r * k_r} \,. \tag{S7}$$

β measures the ratio between all sources contributing to IRF4 induction versus sources contributing to IRF4 degradation, and its numerical value defines regions of bistability, where steady states corresponding to GC B cells and differentiated B cells can coexist[1].

Equation S7 provides the mathematical basis to model B cell differentiation in our updated model. If we further assume that the amount of signaling delivered through the CD40 receptor is constant for cells that bind > 30 min[17], CD40 can be considered a constant in Eq. S7. This amounts to assume that all cells that have received T cell help have received the same amount of CD40 signalling. Under this assumption, all parameters in Eq. S7 are fixed except for the amount of acquired antigen, and hence, β depends linearly on the amount of antigen. Following recent literature that has demonstrated that PCs have significantly higher antigen affinity than memory B cells (MBCs)[16,18], we assume that a CC can leave the GC once it has received T cell help, and the value of β at that time determines whether it becomes a PC or MBC. We can use Eq. S7 and experimental data[19] to find the threshold on β, antigen_threshold, above which a cell differentiate as a PC (Table 1, main text). Hence, in our model, cells that leave the GC with antigen > antigen_threshold become PCs, otherwise, they differentiate as MBCs.

Possible extensions to the intracellular model

The intracellular model described by Eqs. S1-S3 is a minimal model of the B cell differentiation based on 3 master regulators of the GC, BCL6, BLIMP1 and IRF4, according to a previously published model[1]. However, we have also considered possible extensions of this model that include additional factors with relevant roles for the differentiation of B cells. For instance, BACH2 has been reported to co-bind with BCL6 to repress BLIMP transcription[20], and furthermore, it is highly expressed in the GC B cells prone to enter the memory B cell pool[18]. In view of the relevance of BACH2, we studied two extended models that explicitly account for its dynamics. In Extended Model I, BACH2 co-binds with BCL6 to repress BLIMP1[20]. Furthermore, BACH2 is indirectly repressed by BLIMP1 through the mediation of PAX5[21], which is not included in the model. Hence, Extended Model I can be formulated as:

$$\frac{dp}{dt} = \mu_p + \sigma_p \frac{k_a^2}{k_a^2 + a^2} \frac{k_b^2}{k_b^2 + b^2} + \sigma_p \frac{r^2}{k_r^2 + r^2} - \lambda_p p \,, \tag{S8}$$

$$\frac{db}{dt} = \mu_b + \sigma_b \frac{k_p^2}{k_p^2 + p^2} \frac{k_b^2}{k_b^2 + b^2} \frac{k_r^2}{k_r^2 + r^2} - (\lambda_b + bcr)\, b \,, \tag{S9}$$

$$\frac{dr}{dt} = \mu_r + \sigma_r \frac{r^2}{k_r^2 + r^2} + bcr + cd40 - \lambda_r r \tag{S10}$$

$$\frac{da}{dt} = \mu_a + \sigma_a \frac{k_p^2}{k_p^2 + p^2} - \lambda_a a \,, \tag{S11}$$

$$\tag{S12}$$

where a, μ_a, σ_a and λ_a stand respectively for the levels, basal transcription, repression by BLIMP1 and degradation of BACH2. Extended Model I is simulated in Figure S1.

In parallel, we also consider Extended Model II, where in addition to the interactions included in Extended Model I, BACH2

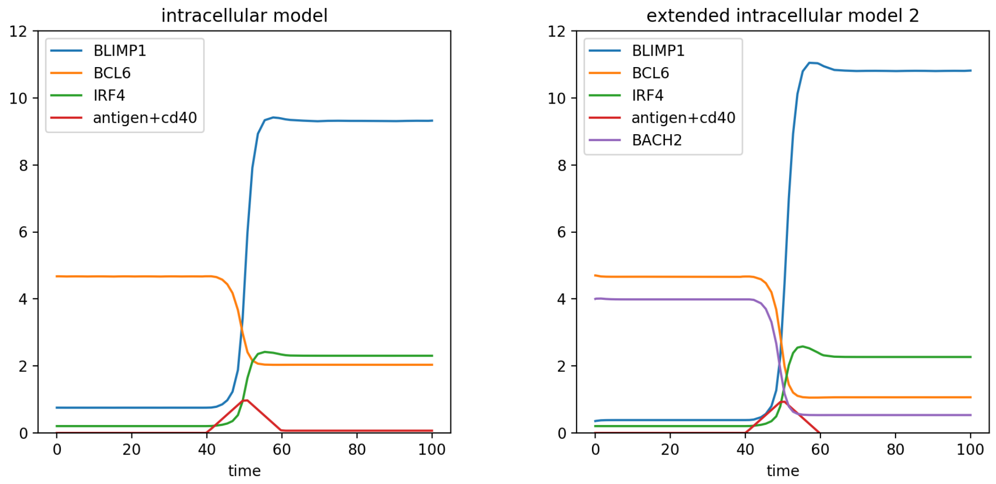

Figure S1. Simulation of Extended Model I. This extended model explicitly includes BACH2, which promotes BLIMP1 transcriptional repression and is highly expressed in the CCs prone to the memory B cell pool. As evident in the figure, BACH2 closely mimics BCL6 expression levels without significantly modifying the pre- and post-signaling steady states of BCL6, BLIMP1 and IRF4. Hence, this model recapitulates experimental evidence without adding new information.

is upregulated by BCL6[22]:

$$\frac{dp}{dt} = \mu_p + \sigma_p \frac{k_a^2}{k_a^2 + a^2} \frac{k_b^2}{k_b^2 + b^2} + \sigma_p \frac{r^2}{k_r^2 + r^2} - \lambda_p\, p\ , \tag{S13}$$

$$\frac{db}{dt} = \mu_b + \sigma_b \frac{k_p^2}{k_p^2 + p^2} \frac{k_b^2}{k_b^2 + b^2} \frac{k_r^2}{k_r^2 + r^2} - (\lambda_b + bcr)\, b\ , \tag{S14}$$

$$\frac{dr}{dt} = \mu_r + \sigma_r \frac{r^2}{k_r^2 + r^2} + bcr + cd40 - \lambda_r\, r \tag{S15}$$

$$\frac{da}{dt} = \mu_a + \sigma_a \frac{b^2}{k_b^2 + b^2} + \sigma_a \frac{k_p^2}{k_p^2 + p^2} - \lambda_a\, a\ . \tag{S16}$$

$$\tag{S17}$$

This model is simulated in figure S2.

All intracellular models are simulated with parameters described in Table S1. As evident in Figures S1 and S2, stimulation via BCR and CD40 signaling induces a switch in the expression levels of all 4 transcription factors corresponding to a differentiation into a PC. However, because BACH2 mirrors both the expression level and the transcriptional activity of BCL6 in both Extended Models, there is no information gain with respect to the minimal model presented in Eqs. S1-S3.

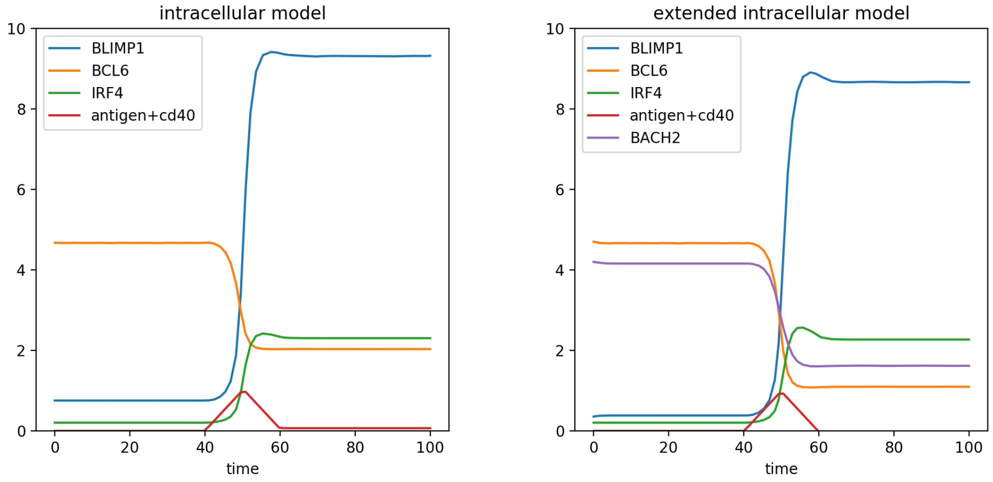

Figure S2. Simulation of Extended Model II. In addition to the interactions described in Extended Model I, BCL6 upregulates BACH2. Similarly to the previous extended model, this model recapitulates experimental evidence without adding additional information.

Parameter	Minimal Model	Extended Model I	Extended Model II
μ_p	$1 * 10^{-6}$	$1 * 10^{-6}$	$1 * 10^{-6}$
μ_b	2	1	1
μ_r	0.1	0.1	0.1
μ_a	-	0.5	0.5
σ_p	9	9	9
σ_b	10	10	10
σ_r	2.6	2.6	2.6
σ_a	-	4	2
$\forall k$	1	1	1
$\forall \lambda$	1	1	1

Table S1. Parameters used in the intracellular model simulations in Figures S1 and S2. The parameters for the minimal model were taken from Martínez et al.[1]. To avoid simply doubling the transcriptional silencing of BLIMP1, we reduced the transcription basal rate of BCL6 and BACH2 in the extended models. According to our simulations, no parameter choice for BACH2 could induce qualitative changes in the model dynamics.

Return time derivation

The simplest and most common model of protein production and degradation is given by:

$$\frac{dx}{dt} = a - x * b,$$

where x is the protein concentration, a is the rate of protein production, and b is the protein degradation. Assuming that both a and b are constant, the steady state is given by $\frac{a}{b}$.

In this model, the protein levels approach their homeostatic value asymptotically and, therefore, the return time to steady state takes a theoretical infinite amount of time. Therefore, return time is commonly defined as a return to a window between 90% and 110% of the steady state. The return time from an arbitrary state x_0 to the nearest edge of the window around the steady state, x_{final}, can be computed as:

$$t = \frac{1}{b} * \log\left(\frac{a - b * x_0}{a - b * x_{final}}\right).$$

We used the above equation to compute the return time to steady state for BCL6 following an asymmetric cell division. We consider the most extreme situation, in which a daughter centroblast (CB) has been left with no BCL6 after a division, i.e. $x_0 = 0$, and has to return to $x_{final} = 0.9 * \frac{a}{b}$. We also consider the symmetric scenario, i.e. a daughter CB has inherited half the available amount of BCL6, i.e. $x_0 = \frac{a}{b}$, and has to return to $x_{final} = 1.1 * \frac{a}{b}$. In both cases, the return time can be estimated to be:

$$t_{return} = \frac{1}{b} * \log(10).$$

b has been estimated to be $\sim$ 4-6 hours for BCL6[23], leading to:

$$t_{return} = 0.46h.$$

Estimation of the time from a CB division to B cell differentiation

We can estimate this time using the rates from the fitted model parameters and by noticing that the expected time until a reaction fires is $\tau \sim (\text{rate})^{-1}$. Hence:

$$\begin{aligned}\tau_{\text{differentiation}} &= E_\tau(\text{CB} \xrightarrow{\text{r}_{\text{migration}}} \text{CC}_{\text{apoptotic}}) \\ &+ E_\tau(\text{CC}_{\text{apoptotic}} + \text{TC} \xrightarrow{\text{r}_{\text{T cell encounter}}} [\text{CCTC}]) \\ &+ E_\tau([\text{CCTC}] \xrightarrow{\text{r}_{\text{activation}}} \text{CC} + \text{TC}) \\ &+ E_\tau(\text{CC} \xrightarrow{\text{r}_{\text{exit}}} \text{PC}) \\ &= 7.69\text{h} + 0.52\text{h} + 0.83\text{h} + 0.45\text{h} = 9.49\text{h}.\end{aligned}$$

The parameters found in the literature lead to an even larger differentiation time:

$$\tau_{\text{differentiation}} = 5.88\text{h} + 14.28\text{h} + 0.50\text{h} + 0.74\text{h} = 21.40\text{h}.$$

While some of these parameters may be inaccurate, the estimated time to differentiation is $\sim$ 20 (47 for the literature parameters) times larger than the relaxation time.

Derivation of parameters from the literature

Each of the ten model parameters is estimated from evidence gathered from the literature.

- $\mathbf{r_{division}}$: Even though the CB division rate has been studied extensively, there is no consensus about its value. Victora and colleagues [2] reviewed several papers[24–27] that placed the cell cycle time between 6 and 12 hours. Meyer et al.[28] assumed a cell division frequency of nine hours. We take an intermediate value from these estimations and assume a centroblast divides every $\sim$ 9h.

The Gillespie algorithm assumes constant reaction rates, i.e. a reaction fires with the same probability at any time. Hence, the time between two subsequent events follows an exponential distribution (this distribution models events that occur continuously and independently at a constant average rate) with rate parameter $\lambda = \mathrm{r_{division}}$:

$$\mathrm{P_{division}} = \lambda * \exp(-\lambda * x) .$$

The mean waiting time between two CB divisions can be estimated as the mean value of the above distribution:

$$< \mathrm{t_{division}} >= \int_0^\infty \lambda * x * \exp(-\lambda * x)\, dx = \frac{1}{\lambda} = 9\,\mathrm{h},$$

from where $\mathrm{r_{division}}$, the probability per unit time that a CB divides, can be estimated to be $\frac{1}{9}\,\mathrm{h}^{-1}$.

- $\mathbf{p_{mutation}}$: The mutation frequency during SHM has been estimated as $\frac{1}{1000}$ per base pair[29]. The variable region, which is the DNA region that defines the shape of the BCR, measures around 500 base pairs[2]. From that, we can calculate the probability of at least one mutation occurring as:

$$P(N_{mut} > 0) = 1 - P(N_{mut} = 0) = 1 - (1 - \frac{1}{1000})^{500} = 0.39 .$$

The above probability does not take into consideration synonymous mutations or mutations that do not change the structure of the BCR significantly, and hence, should be interpreted as an upper bound for the rate of mutations that impact antigen affinity. Meyer-Hermann and colleagues[28] assumed a frequency of 50%, but subsequently tried a larger range of possible parameters in[17].

- $\mathbf{r_{migration}}$: Victora et al. reported that within four hours, half of the CBs in the dark zone have migrated to the light zone[30]. Inserting these values into the cumulative distribution of an exponential distribution yields:

$$1 - \exp(-\lambda * 4\,\mathrm{h}) = 0.5$$
$$\Rightarrow \lambda = \mathrm{r_{migration}} = 0.17\,\mathrm{h}^{-1}.$$

- $\mathbf{r_{apoptosis}}$: The average time for CC to die through apoptosis has been reported to be $\mathrm{E(T_{apoptosis})} = 10$ h[28]. This yields $\mathrm{r_{apoptosis}} = 0.1\ \mathrm{h}^{-1}$.

- $\mathbf{r_{FDC\ encounter}}$: The FDC encounter rate can be derived as follows:

$$\mathrm{r_{FDR\ encounter}} = \mathrm{N_{FDC}} * (\mathrm{v_{CC}} + \mathrm{v_{FDC}}) * \mathrm{A_{effect}} / \mathrm{Vol_{GC}} .$$

The rate of encounter is the volume swept by a CC per unit time multiplied by the average density of FDCs in the GC. More specifically, the effective volume swept by a CC (in relation to the also moving FDCs) is $(\mathrm{v_{CC}} + \mathrm{v_{FDC}}) * \mathrm{A_{effect}}$, where $\mathrm{A_{effect}}$ is the effective area covered by a CC. The density of CCs can be estimated as $\mathrm{N_{FDC}}/\mathrm{Vol_{GC}}$. The parameters for the above equations can be taken from[28]:

$$\mathrm{N_{FDC}} = 10,$$
$$\mathrm{v_{FDC}} = 0,$$
$$\mathrm{v_{CC}} = 5\,\mu\mathrm{m}/\min,$$
$$\mathrm{Vol_{GC}} = \frac{4}{3} * \pi * (220 * \mu\mathrm{m})^3,$$
$$\mathrm{A_{effect}} = 314\,\mu\mathrm{m}^2,$$

where the effective area, $\mathrm{A_{effect}}$, has been derived using an average lymphocyte diameter of $10\,\mu\mathrm{m}$[31], leading to an area $\pi * 10^2\,\mu m^2$. This leads to $\mathrm{r_{FDC\ encounter}} = 0.021\mathrm{h}^{-1}$.

- $\mathbf{r_{T\ cell\ encounter}}$: The T cell encounter rate is computed analogously to $\mathrm{r_{FDC\ encounter}}$. We use $\mathrm{v_{TC}} = 10.8\,\mu\mathrm{m}/\min$[28] and $\mathrm{N_{TC}} = 10$[17,28]. This results in $\mathrm{r_{T\ cell\ encounter}} = 0.067\ \mathrm{h}^{-1}$.

- $\mathbf{r_{activation}}$: Since the model of T cell help was taken from Meyer-Hermann and colleagues[17], we also take a CC-TC binding time of 30 min from this reference, leading to $\mathrm{r_{activation}} = 2\ \mathrm{h}^{-1}$.

- $\mathbf{r_{exit}}$: Meyer-Hermann et al. modeled cell exit as a chemotaxis process[17]. Using their parameter values[28], we can compute $\mathrm{r_{exit}}$ as follows:

$$\mathrm{t_{exit}} = \frac{r_{GC}}{v_{CC}} = \frac{220\,\mu\mathrm{m}}{5\,\mu\mathrm{m/min}} = 44\mathrm{min}.$$
$$\Rightarrow \mathrm{r_{exit}} = \frac{1}{44}\mathrm{min}^{-1} = 1.36\mathrm{h}^{-1}.$$

- $\mathbf{r_{recirculation}}$: Victora et al. found that around 30% of centroblasts return to the dark zone after selection in the light zone[30], which can be formulated as:

$$\mathrm{P(t_{recirculation} < t_{exit})} = 1 - \exp(-\lambda * \mathrm{t_{exit}}) = 0.3\,,$$

using the previously computed $\mathrm{t_{exit}}$, we get $\lambda = \mathrm{r_{recirculation}} = 0.49\ \mathrm{h}^{-1}$.

- **antigen_threshold**: The antigen threshold to differentiate into a PC is a novel concept introduced in this paper, and therefore, there is no experimental data available to compute it. In this paper, we optimize this value using time series data of the ratio of MBCs to PCs produced by the GC, kindly provided by Weisel et al.[19].

Gillespie algorithm with continuous particle properties

We present here a two-steps algorithm to perform stochastic simulations based on the Gillespie algorithm, while simultaneously keeping track of individual particle properties. As described in the main text, our approach does not need discretization, and hence, does not result in loss of accuracy.

Let us assume a system with the states A, B, where each particle in state A or B has an individual property i. To use a traditional Gillespie algorithm, such properties would need to be discretized into species $A_0, A_1, \ldots A_n$ and $B_0, B_1, \ldots B_n$. As a toy example, let us assume that the system has the following reaction channels:

$$\mathrm{A}_i^2 \xrightarrow{\mathrm{r_1(i_1,i_2)}} \mathrm{B}^2_{\mathrm{i+const_3}} \tag{S18}$$
$$\mathrm{B}_i \xrightarrow{\mathrm{r_2}} \mathrm{A}_i, \tag{S19}$$

where

$$r_1(i_1,i_2) = \frac{i_1 * i_2}{i_1 * i_2 + const_1}$$
$$r_2 = const_2\,,$$

and i is increased by $\mathrm{const_3}$ after $\mathrm{r_1}$.

The system can be simulated in two steps:

1. Individual properties are ignored. The reaction rate of each reaction channel is replaced by an upper bound $r_{k,max}$, which can either be the highest reaction rate for particles currently in the simulation or a static upper bound. In our example, since $r_{1,max}$ is bounded by 1, we take $r_{1,max} = 1$. Regarding r_2, since this rate is independent of i, its upper boundary is simply $\mathrm{const_2}$. This results in a simplified system, as follows:

$$\mathrm{A}^2 \xrightarrow{\mathrm{r_{1,max}}} \mathrm{B}^2$$
$$\mathrm{B} \xrightarrow{\mathrm{const_2}} \mathrm{A}.$$

   Next, the simplified system is simulated using the classical Gillespie algorithm[32]. This entails calculating the propensities p from the reaction rates and the numbers of particles N:

$$p_{1,\mathrm{max}} = \frac{N_A * (N_A - 1)}{2} * r_{1,\mathrm{max}}$$
$$p_2 = N_B * r_2$$

and drawing a reaction channel k and a time τ:

$$k = \min_k \left(\sum_{i=1}^{k} p_i > U_0^{\sum p} \right)$$

$$\tau = -\frac{\log(U_0^1)}{\sum p} ,$$

where U_a^b are uniform random numbers in the ranges $[a, b)$.

2. The system's time may already be advanced by τ, but the individual properties i must be accounted for before changing the species' counts. To do that, we first draw individual properties i from a particle list in the form of:

$$A : \{i_1, i_2, i_3, ..., i_{N_A}\}$$
$$B : \{i_1, i_2, i_3, ..., i_{N_B}\}.$$

Each i in the list represents the individual properties of exactly one particle in state A or B. Next, for each reactant in the drawn reaction k, we select one i from the respective list with a uniform probability. For instance, for $k = 1$, we would draw two i's from list A, as reaction 1 requires two particles A to interact. In this case, attention should be paid not to draw the same particle twice. Let us call the drawn i's i_m and i_n. The true propensity of reaction 1 can be computed as:

$$p_{1,\text{true}} = \frac{N_A * (N_A - 1)}{2} * \frac{i_m * i_n}{i_m * i_n + const_1} . \tag{S20}$$

We compare this propensity to $p_{1,max}$ by constructing an acceptance probability:

$$P_{\text{acceptance}} = \frac{p_{k,\text{true}}}{p_{k,\text{max}}} .$$

We draw a new random random U_0^1, and proceed with the reaction only if:

$$U_0^1 < P_{\text{acceptance}} ,$$

otherwise, the reaction is rejected and the cell counts are not changed. Notice that the decision on whether the reaction takes places or not is done by using the true propensity given by Eq. S20, and hence, each reaction with each possible combination of individual particles happens with their exact true probability.

If the reaction is accepted, the reaction counts are changed and the particle lists are modified according to the reaction. In our example with $k = 1$, we would remove i_m and i_n from list A and append i_m + $const_3$ and i_n + $const_3$ to list B, according to Eq. S18.

An alternative formulation of the reaction propensities where the rejected reactions are made explicit is:

$$\mathrm{A}_i^2 \xrightarrow{\mathrm{r_1(i_1,i_2)}} \mathrm{B}^2_{\mathrm{i+const_3}} ,$$
$$\mathrm{B}_i \xrightarrow{\mathrm{r_2}} \mathrm{A}_i ,$$
$$\emptyset \xrightarrow{\mathrm{r_{rejection}}} \emptyset ,$$

with a propensity for the rejected reactions given by:

$$p_{\text{rejection}} = \sum_{i \in A \setminus j} \sum_{j \in A} \left(r_{1,\text{max}} - \frac{i * j}{i * j + const_1} \right) .$$

Since all 3 reactions are accounted for in the calculation of τ, this is equivalent to adding an empty reaction channel where no particle count gets updated, and hence, whose outcome does not influence the system's evolution. The Gillespie algorithm is agnostic to such independent reactions, since all reactions preserve their reaction rate when adding more reaction channels.

Deterministic analysis

Since a stochastic stability analysis can only be performed locally, we also perform a deterministic stability analysis to gain insight about the effect of parameter changes on the model behavior. For the sake of a stability analysis, two reactions can be neglected in the deterministic model:

- The substitution reaction:

$$[\mathrm{CC_1TC}] + \mathrm{CC_{2,apoptotic}} + \xrightarrow{\mathrm{r_{T\ cell\ encounter}}} \mathrm{CC_{1,apototic}} + [\mathrm{CC_2TC}]\,,$$

  since this reaction only swaps individual particles in the same population, and hence, does not affect the population dynamics.

- The FDC – CC encounter reaction:

$$\mathrm{CC_{apoptotic}} \xrightarrow{\mathrm{r_{FDC\ encounter}}} \mathrm{CC_{apoptotic}}\,.$$

  Similarly, this reaction might increase the amount of antigen in a CC, but does not change the population dynamics.

This leads to the following deterministic system:

$$\frac{d\,CB}{dt} = r_{\mathrm{div}} * CB - r_{\mathrm{migr}} * CB + r_{\mathrm{recirc}} * CC_{\mathrm{apop}}\,, \tag{S21}$$

$$\frac{d\,CC_{\mathrm{apop}}}{dt} = r_{\mathrm{migr}} * CB - r_{\mathrm{apop}} * CC_{\mathrm{apop}} - r_{\mathrm{TC\ enc}} * TC * CC_{\mathrm{apop}} \tag{S22}$$

$$\frac{d\,[CCTC]}{dt} = r_{\mathrm{TC\ enc}} * TC * CC_{\mathrm{apop}} - [CCTC] * r_{\mathrm{act}} \tag{S23}$$

$$\frac{d\,CC}{dt} = [CCTC] * r_{\mathrm{act}} - CC * (r_{\mathrm{exit}} + r_{\mathrm{recirc}})\,. \tag{S24}$$

In addition, solutions to this system of ODEs must satisfy the algebraic constraint TC + [CCTC] = $\mathrm{TC_{total}}$= const., which states that the number of TC cells remains constant through our simulation. The deterministic steady state can be found by setting the time evolution of Eqs. S21-S24 to zero. We require the resulting steady states to be non-negative using the constraints:

$$\forall\ \text{rates} > 0$$
$$\forall\ \text{steady states} \geq 0\,.$$

This leads to the following parameter bounds for systems with physical, nonzero steady states:

$$\mathrm{r_{migr}} > \mathrm{r_{div}}$$
$$1 > \left(1 + \frac{\mathrm{r_{apop}}}{\mathrm{r_{TC\ enc}} * \mathrm{TC_{total}}}\right) * \left(1 + \frac{\mathrm{r_{exit}}}{\mathrm{r_{recirc}}}\right) * \left(1 - \frac{\mathrm{r_{div}}}{\mathrm{r_{migr}}}\right)\,.$$

We also performed a Jacobian stability analysis[33]. The analysis of the Jacobian matrix results in a quartic characteristic polynomial, which symbolic solvers such as Mathematica[34] are unable to solve in a closed form. We also used the Routh-Hurwitz stability criterion[33], which can identify necessary and sufficient conditions for stability in a linearized, time-invariant system. However, the resulting stability criteria were either redundant with the constraints we already found, or they did not resolve analytically. We therefore rely on the numerical stochastic stability analysis described in the main paper to determine the bounds of the stable regime.

Supplementary figures

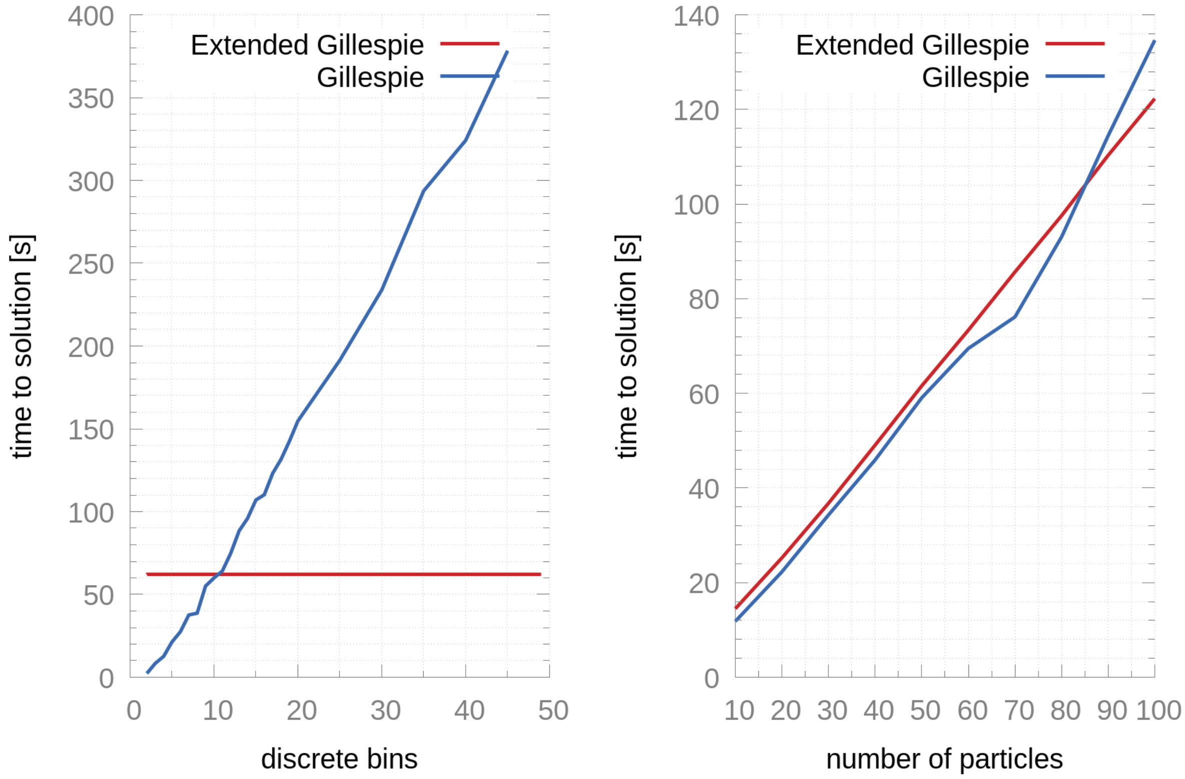

Figure S3. Performance comparison between the novel algorithm and the traditional Gillespie. The novel algorithm offers machine precision using the same amount of time as a traditional simulation with only ten discrete bins. On the left, the number of particles is set to 50, and we investigate the running time of both algorithms for increasingly larger numbers of discretization bins in the traditional Gillespie algorithm. On the right, we explore the increase in running times as the number of particles increases. To ensure a fair comparison, we set the traditional Gillespie to 10 discretization bins. Both algorithms perform similarly, meaning that the novel algorithm maintains the same complexity as the traditional Gillespie algorithm, and the larger memory footprint had no negative impact on performance. The simulated system is described in the SI section '*Gillespie algorithm with continuous particle properties*'.

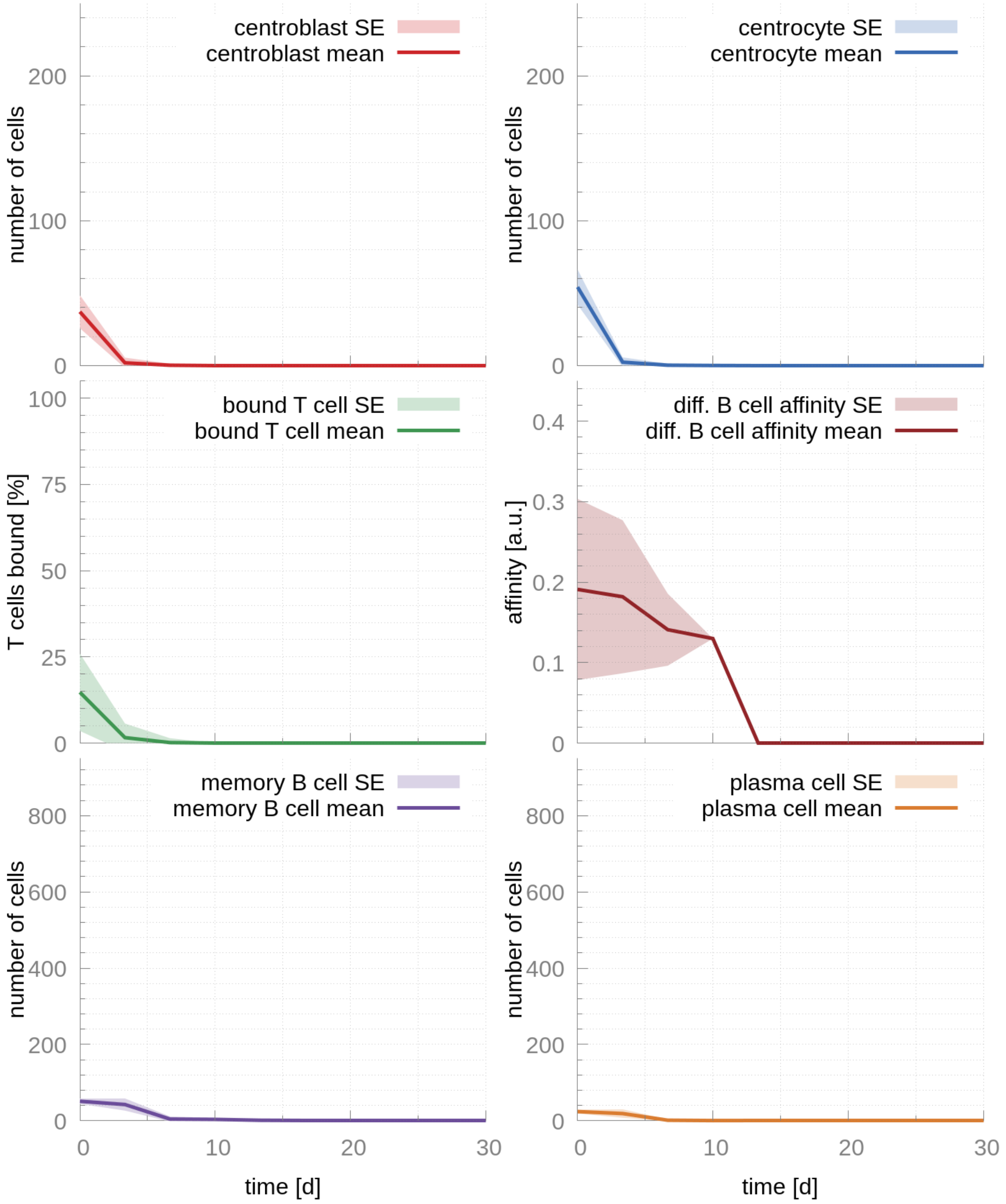

Figure S4. Simulations of the GC output and affinity maturation using unmodified literature parameters. The simulations were performed using the parameters derived from experimental bounds (literature parameters) without any additional adjustment. The parameters found in the literature before the manual adjustments. As evident from the simulations, all cell populations quickly go to zero, and hence we conclude that this set of parameters does not result in a stable GC. The shaded areas depict the standard error (SE)

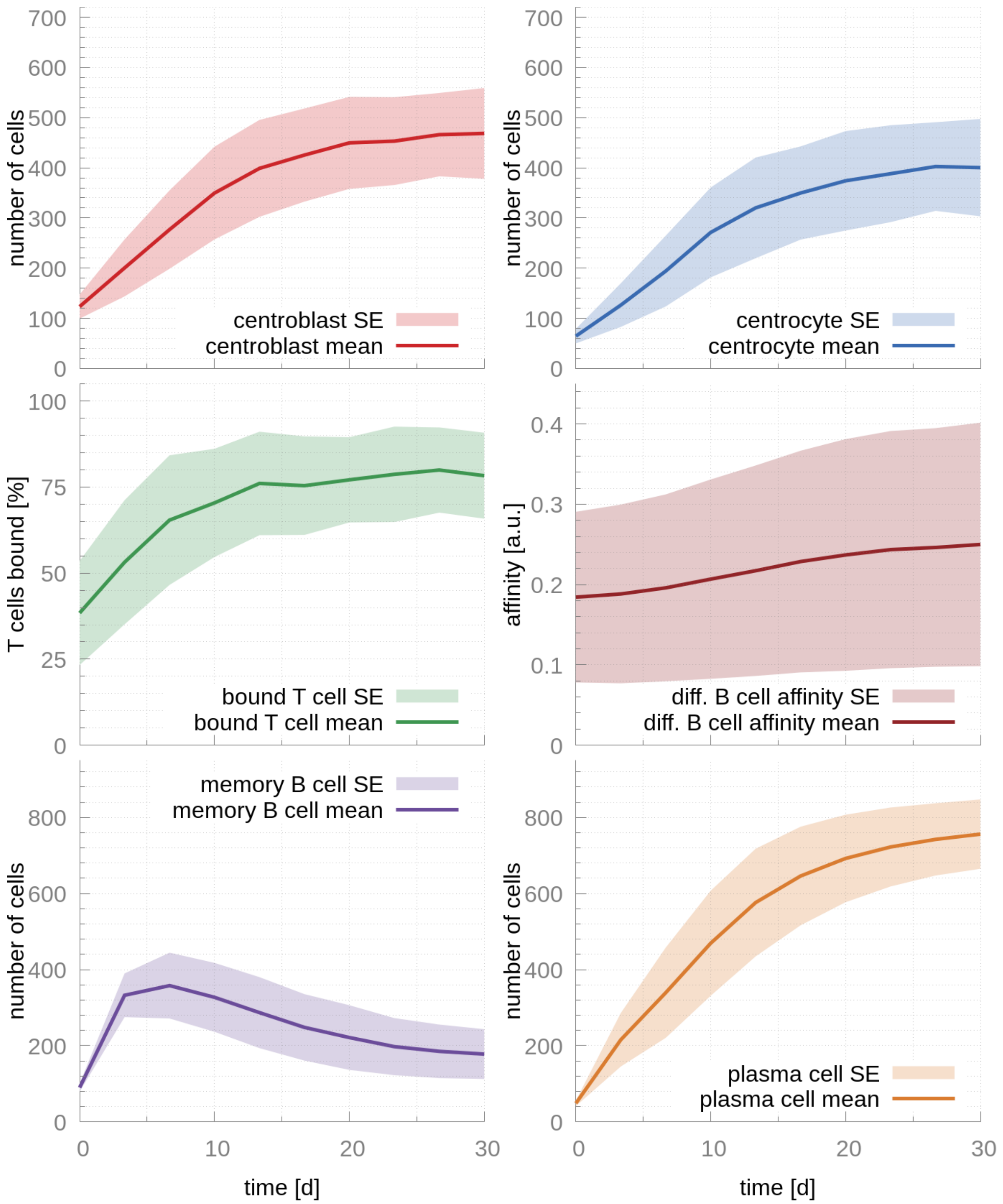

Figure S5. Simulations of the GC output and affinity maturation using modified literature parameters. The stabilized literature parameters in the Figure 2 of the main paper did not exhibit affinity maturation. In this simulation, we increased $r_{\text{T cell encounter}}$ threefold. The resulting simulation displays a higher TC occupation rate of up to 80%. Consequently, the system also exhibits affinity maturation and a switch from MBC to PC production. The shaded areas depict the standard error (SE)

References

1. Martínez, M. R. *et al.* Quantitative modeling of the terminal differentiation of b cells and mechanisms of lymphomagenesis. *Proc. Natl. Acad. Sci.* **109**, 2672–2677 (2012).
2. Victora, G. D. & Nussenzweig, M. C. Germinal centers. *Annu. review immunology* **30**, 429–457 (2012).
3. Klein, U. & Dalla-Favera, R. Germinal centres: role in b-cell physiology and malignancy. *Nat. reviews. Immunol.* **8**, 22 (2008).
4. Basso, K. & Dalla-Favera, R. Bcl6: master regulator of the germinal center reaction and key oncogene in b cell lymphomagenesis. *Adv. immunology* **105**, 193–210 (2010).
5. Bihui, H. Y. *et al.* The bcl-6 proto-oncogene controls germinal-centre formation and th2-type inflammation. *Nat. genetics* **16**, 161–170 (1997).
6. Dent, A. L., Shaffer, A. L., Yu, X., Allman, D. & Staudt, L. M. Control of inflammation, cytokine expression, and germinal center formation by bcl-6. *Science* **276**, 589–592 (1997).
7. De Silva, N. S., Simonetti, G., Heise, N. & Klein, U. The diverse roles of irf4 in late germinal center b-cell differentiation. *Immunol. Rev.* **247**, 73–92, DOI: 10.1111/j.1600-065X.2012.01113.x (2012).
8. Ranuncolo, S. M. *et al.* Bcl-6 mediates the germinal center b cell phenotype and lymphomagenesis through transcriptional repression of the dna-damage sensor atr. *Nat. immunology* **8**, 705 (2007).
9. Shaffer, A. *et al.* Bcl-6 represses genes that function in lymphocyte differentiation, inflammation, and cell cycle control. *Immunity* **13**, 199–212 (2000).
10. Tunyaplin, C. *et al.* Direct repression of prdm1 by bcl-6 inhibits plasmacytic differentiation. *The J. Immunol.* **173**, 1158–1165 (2004).
11. Vasanwala, F. H., Kusam, S., Toney, L. M. & Dent, A. L. Repression of ap-1 function: a mechanism for the regulation of blimp-1 expression and b lymphocyte differentiation by the b cell lymphoma-6 protooncogene. *The J. Immunol.* **169**, 1922–1929 (2002).
12. Fyfe, G. *et al.* Subpopulations of b lymphocytes in germinal centers. *The J. Immunol.* **139**, 2187–2194 (1987).
13. Turner, C. A., Mack, D. H. & Davis, M. M. Blimp-1, a novel zinc finger-containing protein that can drive the maturation of b lymphocytes into immunoglobulin-secreting cells. *Cell* **77**, 297–306 (1994).
14. Shapiro-Shelef, M. *et al.* Blimp-1 is required for the formation of immunoglobulin secreting plasma cells and pre-plasma memory b cells. *Immunity* **19**, 607–620 (2003).
15. Sciammas, R. *et al.* An incoherent regulatory network architecture that orchestrates b cell diversification in response to antigen signaling. *Mol. systems biology* **7**, 495 (2011).
16. Ochiai, K. *et al.* Transcriptional regulation of germinal center b and plasma cell fates by dynamical control of {IRF4}. *Immunity* **38**, 918 – 929, DOI: https://doi.org/10.1016/j.immuni.2013.04.009 (2013).
17. Meyer-Hermann, M. *et al.* A theory of germinal center b cell selection, division, and exit. *Cell Reports* **2**, 162 – 174, DOI: https://doi.org/10.1016/j.celrep.2012.05.010 (2012).
18. Shinnakasu, R. *et al.* Regulated selection of germinal-center cells into the memory b cell compartment. *Nat. immunology* **17**, 861–872 (2016).
19. Weisel, F. J., Zuccarino-Catania, G. V., Chikina, M. & Shlomchik, M. J. A temporal switch in the germinal center determines differential output of memory b and plasma cells. *Immunity* **44**, 116–130 (2016).
20. Huang, C., Geng, H., Boss, I., Wang, L. & Melnick, A. Cooperative transcriptional repression by BCL6 and BACH2 in germinal center B-cell differentiation. *Blood* **123**, 1012–1020, DOI: 10.1182/blood-2013-07-518605 (2014).
21. Muto, A. *et al.* Bach2 represses plasma cell gene regulatory network in b cells to promote antibody class switch. *The EMBO journal* **29**, 4048–4061 (2010).
22. Alinikula, J., Nera, K.-P., Junttila, S. & Lassila, O. Alternate pathways for bcl6-mediated regulation of b cell to plasma cell differentiation. *Eur. journal immunology* **41**, 2404–2413 (2011).
23. Niu, H., Ye, B. H. & Dalla-Favera, R. Antigen receptor signaling induces MAP kinase-mediated phosphorylation and degradation of the BCL-6 transcription factor. *Genes & Dev.* **12**, 1953–1961, DOI: 10.1101/gad.12.13.1953 (1998).
24. Allen, C. D., Okada, T., Tang, H. L. & Cyster, J. G. Imaging of germinal center selection events during affinity maturation. *Science* **315**, 528–531 (2007).

25. Hauser, A. E. *et al.* Definition of germinal-center b cell migration in vivo reveals predominant intrazonal circulation patterns. *Immunity* **26**, 655–667 (2007).

26. Zhang, J., MacLennan, I. C., Liu, Y.-J. & Lane, P. J. Is rapid proliferation in b centroblasts linked to somatic mutation in memory b cell clones? *Immunol. letters* **18**, 297–299 (1988).

27. Liu, Y.-J., Zhang, J., Lane, P. J., Chan, E. Y.-T. & Maclennan, I. Sites of specific b cell activation in primary and secondary responses to t cell-dependent and t cell-independent antigens. *Eur. journal immunology* **21**, 2951–2962 (1991).

28. Meyer-Hermann, M. E., Maini, P. K. & Iber, D. An analysis of b cell selection mechanisms in germinal centers. *Math. Medicine Biol.* **23**, 255–277 (2006).

29. Di Noia, J. M. & Neuberger, M. S. Molecular mechanisms of antibody somatic hypermutation. *Annu. Rev. Biochem.* **76**, 1–22 (2007).

30. Victora, G. D. *et al.* Germinal center dynamics revealed by multiphoton microscopy with a photoactivatable fluorescent reporter. *Cell* **143**, 592–605 (2010).

31. Dixon, J. B., Gashev, A. A., Zawieja, D. C., Moore, J. E. & Coté, G. L. Image correlation algorithm for measuring lymphocyte velocity and diameter changes in contracting microlymphatics. *Annals biomedical engineering* **35**, 387–396 (2007).

32. Gillespie, D. T. Exact stochastic simulation of coupled chemical reactions. *The journal physical chemistry* **81**, 2340–2361 (1977).

33. Guzzella, L. *Analysis and synthesis of Single-input Single-output control systems* (vdf Hochschulverlag AG, 2011).

34. Inc., W. R. Mathematica, Version 11.1. Champaign, IL, 2017.
